# Metabolic collusion driving immune evasion in cholangiocarcinoma: unmasking the dual control of the immuno-metabolic microenvironment

**DOI:** 10.3389/fimmu.2025.1697056

**Published:** 2025-11-28

**Authors:** Jingnan Xue, Longhao Zhang, Kai Zhang, Yu Wu, Kai Zhou, Xin Lu

**Affiliations:** 1 Department of Hepatobiliary Surgery, People’s Hospital of Anshun City, Guizhou, China; 2 Department of Liver Surgery, Peking Union Medical College Hospital, Chinese Academy of Medical Sciences & Peking Union Medical College, Beijing, China

**Keywords:** cholangiocarcinoma, immune evasion, metabolic reprogramming, tumor microenvironment, bile acids, combination immunotherapy

## Abstract

Cholangiocarcinoma represents an aggressive malignancy with poor prognosis, particularly for intrahepatic Cholangiocarcinoma. Despite recent advancements in chemotherapy and immune checkpoint blockade therapies, survival outcomes remain suboptimal. A key obstacle in treating Cholangiocarcinoma is its immune exclusion and resistance to Cholangiocarcinoma, which is influenced by metabolic reprogramming within the tumor microenvironment. This review explores the dual control of metabolism and immunity in Cholangiocarcinoma, highlighting the intricate interplay between metabolic pathways (e.g., glycolysis, lactate accumulation, fatty acid oxidation) and immune evasion mechanisms. We examine how bile acid signaling, hypoxia, and stromal interactions shape Cholangiocarcinoma’s immune landscape, facilitating tumor progression and immune resistance. Moreover, we discuss emerging therapeutic strategies that target metabolic vulnerabilities to “convert” immune-excluded Cholangiocarcinoma into a more immunologically responsive state. These strategies include metabolic inhibitors targeting lactate, amino acid catabolism, and fatty acid metabolism, as well as approaches to modulate bile acid signaling. We propose that combining metabolic reprogramming with immune checkpoint blockade therapies holds significant promise in enhancing immune responses and improving therapeutic outcomes for Cholangiocarcinoma patients. This review provides a comprehensive framework for future research and clinical trials, aiming to bridge the gap between metabolic insights and immunotherapy in Cholangiocarcinoma treatment.

## 1 Introduction

Cholangiocarcinoma (CCA)—arising from the intrahepatic and extrahepatic biliary tree—remains an aggressive malignancy with increasing incidence in many regions and persistently poor survival outcomes, particularly for intrahepatic CCA (iCCA). Contemporary population studies and national cohorts confirm rising rates and high mortality despite modest therapeutic gains, underscoring a substantial global disease burden and unmet clinical need (1–3). For advanced biliary tract cancer (BTC), gemcitabine–cisplatin had long represented the first-line standard. Recently, adding immune checkpoint blockade (ICB) to chemotherapy has produced statistically significant, practice-changing but still incremental survival benefits. In TOPAZ-1, durvalumab plus gemcitabine–cisplatin improved median overall survival (OS) versus chemotherapy alone and approximately doubled the 24-month OS rate with longer follow-up; nevertheless, most patients ultimately progress (4). Similarly, KEYNOTE-966 showed a significant OS advantage for pembrolizumab plus gemcitabine–cisplatin over chemotherapy alone without new safety signals (5). These pivotal trials established chemo-immunotherapy as a new standard, yet the magnitude of benefit remains limited for a large fraction of patients.

Converging evidence positions metabolic reprogramming as a core hallmark of cancer biology with direct consequences for anti-tumor immunity. Foundational work has codified metabolic rewiring (e.g., enhanced glycolysis, altered redox and lipid programs) as a cancer hallmark, catalyzing a surge of “immunometabolism” research that links tumor/stromal metabolic states to immune cell fate and function within the tumor immune microenvironment (TIME) (6). Contemporary overviews further emphasize actionable crosstalk between nutrients, metabolites (lactate, kynurenine, adenosine, bile acids), and immune checkpoints/signaling pathways, suggesting therapeutic leverage points for precision combinations (7, 8). CCA exhibits distinctive immuno-metabolic features shaped by biliary physiology: a dense desmoplastic stroma that restricts T-cell trafficking and fosters immune exclusion; and a bile acid–rich biochemical milieu that signals through FXR/TGR5 and related receptors to remodel innate and adaptive immunity (9). These properties help explain why many CCA tumors present “cold” or immune-excluded phenotypes with limited responsiveness to ICB, and they highlight metabolism-immunity nodes as candidates for therapeutic intervention (10).

**Figure 1:**
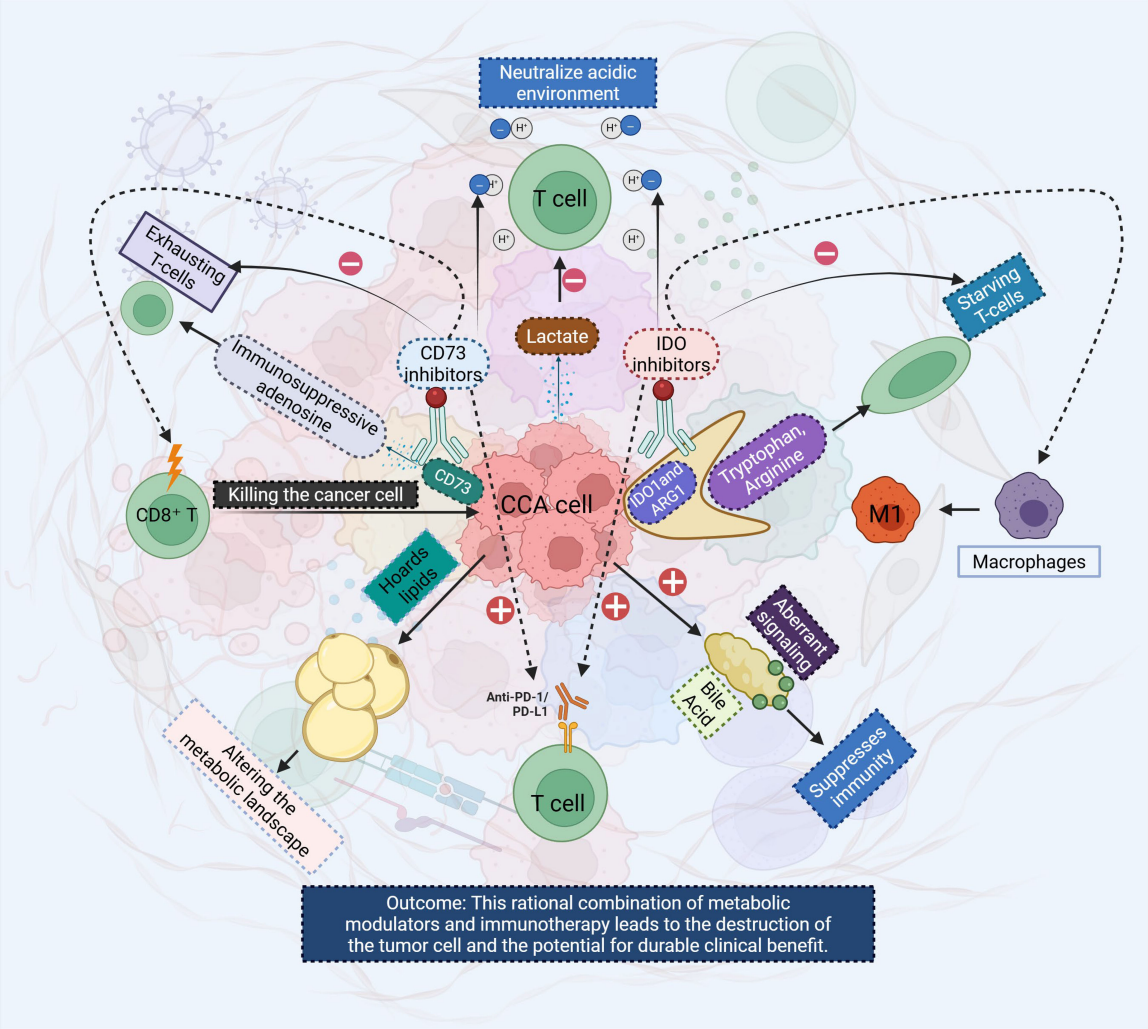
This diagram illustrates the complex interplay between metabolic modulation and immune checkpoint inhibition in the TME of CCA. The CCA cells undergo metabolic reprogramming to hoard lipids and produce lactate, which fosters an acidic environment that suppresses immune cell activity. The figure shows various therapeutic strategies aimed at reversing immune suppression, including CD73 inhibitors, which block the immunosuppressive adenosine pathway, and IDO inhibitors targeting the tryptophan/arginine metabolic axis. The combination of anti-PD-1/PD-L1 antibodies and metabolic modulators is highlighted as a potential strategy to restore T cell function, promote tumor cell killing by CD8+ T cells, and activate macrophages (M1) to enhance antitumor immunity. The outcome is an integrated therapeutic approach that could improve clinical outcomes by altering the metabolic landscape of the tumor and overcoming immune evasion mechanisms. It visually summarizes the core problem (the “cold” tumor) and the proposed solution (metabolism-informed combination immunotherapy to create a “hot” tumor). CCA, Cholangiocarcinoma; TME, tumor microenvironment.

This review synthesizes bidirectional links between metabolism and immune evasion in CCA—how tumor, stromal, and microbial/bile acid–mediated metabolic programs sculpt the TIME, and how immune signaling reciprocally reprograms cellular metabolism. We integrate recent clinical advances in chemo-immunotherapy with mechanistic insights to outline testable, translational strategies (e.g., targeting lactate transport, adenosine generation, amino-acid catabolism, fatty-acid metabolism, or bile acid signaling) aimed at converting “cold” to “hot” CCA and enhancing durable benefit from ICB-based regimens (11–13). By aligning epidemiologic urgency with mechanistic opportunity, the framework presented here seeks to guide rational biomarker development and the design of next-generation, metabolism-informed immunotherapy combinations in BTC (13) (**Figure 1**).

## 2 Molecular and immuno-metabolic landscape of CCA

### 2.1 Anatomical and molecular subtypes

CCA is anatomically classified as intrahepatic (iCCA), perihilar cholangiocarcinoma (pCCA) and distal cholangiocarcinoma (dCCA), each with distinct cells of origin, histology and therapeutic vulnerabilities (14). Within iCCA, a robust pathologic dichotomy separates small-duct and large-duct subtypes: small-duct iCCA (often mass-forming, non-mucinous) is enriched for IDH1/2 mutations and FGFR2 fusions, whereas large-duct iCCA (frequently mucinous, periductal infiltrative) molecularly resembles extrahepatic tumors and is enriched for KRAS, TP53, and SMAD4 alterations (15, 16). In recent immunogenomic studies, the terms “inflamed” and “non-inflamed” refer to distinct immune microenvironmental states within tumors, particularly in CCA (17). “Inflamed” refers to tumors exhibiting active immune infiltration, characterized by the presence of immune cells such as T cells, dendritic cells, and macrophages, along with elevated levels of pro-inflammatory cytokines and chemokines. These tumors often show signs of immune response activation, such as PD-L1 expression and T cell receptor signaling, and tend to respond better to immunotherapies due to the presence of an active immune response. On the other hand, “non-inflamed” tumors are those with sparse immune cell infiltration, low expression of immune checkpoints, and an immunosuppressive microenvironment. These tumors are often more resistant to immunotherapy, as the lack of immune cell activity allows tumor cells to escape immune surveillance. The distinction between these states is crucial for understanding how CCA tumors may respond to immunotherapies and other treatments, and the characterization of these immune landscapes can guide patient stratification and therapeutic decision-making (18).

### 2.2 Driver alterations and pathway wiring

Genomic profiling of CCA has revealed distinct, site-specific patterns of actionable alterations, underscoring the molecular heterogeneity of this malignancy (19, 20). In iCCA, FGFR2 rearrangements and IDH1/2 mutations recur frequently and now anchor approved targeted therapies, making them pivotal biomarkers for patient stratification. Additional common alterations include BAP1 and ARID1A, particularly in the small-duct iCCA subtype, where these changes are often mutually exclusive with KRAS/TP53/SMAD4 driver mutations and correlate with better prognosis. Conversely, extrahepatic cholangiocarcinoma (eCCA) more commonly shows KRAS and TP53 mutations as well as ERBB2 (HER2) amplification/mutation, with actionable frequencies supportive of routine molecular testing. A recent Japanese multi-omics study further identified PRKACA/PRKACB fusions as preferential events in eCCA, and uncovered an APOBEC mutational signature predominating in eCCA and gallbladder carcinoma, highlighting distinct etiologic and mutagenic processes across the biliary tree. These lesion patterns converge on therapeutically tractable pathways—including FGFR, IDH, ERBB, MAPK and PI3K–AKT–mTOR—which are increasingly being integrated into clinical trial design and real-world decision-making in BTC (21, 22). This evolving landscape not only advances targeted therapeutic opportunities but also emphasizes the necessity of anatomical and molecular stratification for precision medicine in CCA (23). As molecular diagnostics become more accessible, the interplay between anatomical subtype, genomic driver, and therapeutic vulnerability will increasingly guide individualized care in advanced disease.

### 2.3 Tissue ecology: bile-acid signaling, hypoxia and desmoplasia

CCA arises within a biochemically unique and highly fibrotic niche. Bile-acid–rich signaling shapes tumor biology and immunity through nuclear and membrane receptors. Preclinical and translational data indicate FXR activation may restrain, whereas TGR5 activation may promote, CCA progression, highlighting receptor-specific and context-dependent effects of the biliary milieu (24, 25). Hypoxia and dense desmoplasia (cancer-associated fibroblasts, CAFs) are hallmark features that remodel metabolism and immune trafficking. Hypoxia–HIF programs correlate with adverse prognosis in CHOL datasets and can rewire lipid and oxidative metabolism in iCCA (26, 27); CAF-rich stroma alters tumor cell bioenergetics, stiffens extracellular matrix, and imposes physical/chemical barriers to effector T-cell infiltration. Recent mechanistic work shows fibroblast-derived factors (e.g., lysyl oxidase, LOX) boost oxidative phosphorylation (OXPHOS) and stemness in CCA cells, while comprehensive reviews catalog CAF heterogeneity and its immunosuppressive crosstalk (e.g., TGF-β–driven programs) (28, 29).

### 2.4 Immune ecology and its coupling to metabolism

Across cohorts of CCA, tumors often present distinct immune phenotypes—namely myeloid-inflamed, T-cell-excluded, or non-inflamed (desert) states—with stromal and metabolic barriers, such as acidic/lactate-rich microenvironments, adenosine elevation, and bile-acid signaling, limiting T-cell priming, infiltration and effector function (30). An immunogenomic analysis of iCCA identified reproducible immune modules—myeloid inflammation/KRAS, antigen-presentation/proliferation, and checkpoint-signaling—that correlate with variable responses to immunotherapy and point toward combinatorial targets at the metabolism–immunity interface. Classical biomarkers of immune-checkpoint inhibitor (ICI) sensitivity remain rare in BTCs: microsatellite instability-high (MSI-H)/deficient mismatch repair (dMMR) occur in only ~1.7-3.1% of cases (31), and high TMB is infrequent and context-dependent—findings that help explain the modest single-agent ICI activity seen and further emphasize the need for metabolism-aware stratagems to convert immune-excluded or desert states into more immune-inflamed ones. Emerging evidence demonstrates that lactate accumulation within hypoxic tumor regions induces histone lactylation and reprograms macrophages toward M2-like phenotypes, adenosine generated via CD73 up-regulation suppresses CD8^+^ T-cells, and bile acids activate CAFs via GPBAR1 to foster neutrophil recruitment and immunosuppressive stroma in CCA (32, 33). These findings together support a model in which metabolism and immune exclusion act in concert to thwart effective immunotherapy, thereby underscoring a translational imperative: combining metabolic modulation (e.g., targeting lactate, adenosine, bile-acid signaling) with immune-checkpoint blockade may overcome CCA’s intrinsic resistance to immunotherapy.

#### 3 Metabolic programs driving immune evasion in CCA

### 3.1 Glycolysis–lactate axis and an acidic tumor milieu

CCA cells display heightened glycolytic flux with extracellular lactate accumulation and pH decline, which directly impairs dendritic-cell maturation, NK-cell cytotoxicity, and CD8^+^ T-cell effector function (34). In CCA models, lactic acidosis augments malignant phenotypes via EGFR–ALDH1A3 signaling, underscoring disease-specific coupling between acid–base stress and oncogenic programs (35). Functionally, blocking the lactate exporter MCT4 can reverse lactate-driven immunosuppression and enhance response to immune-checkpoint blockade in preclinical systems, nominating the lactate–MCT4 axis as a tractable immunometabolic vulnerability (36, 37).

**Figure 2:**
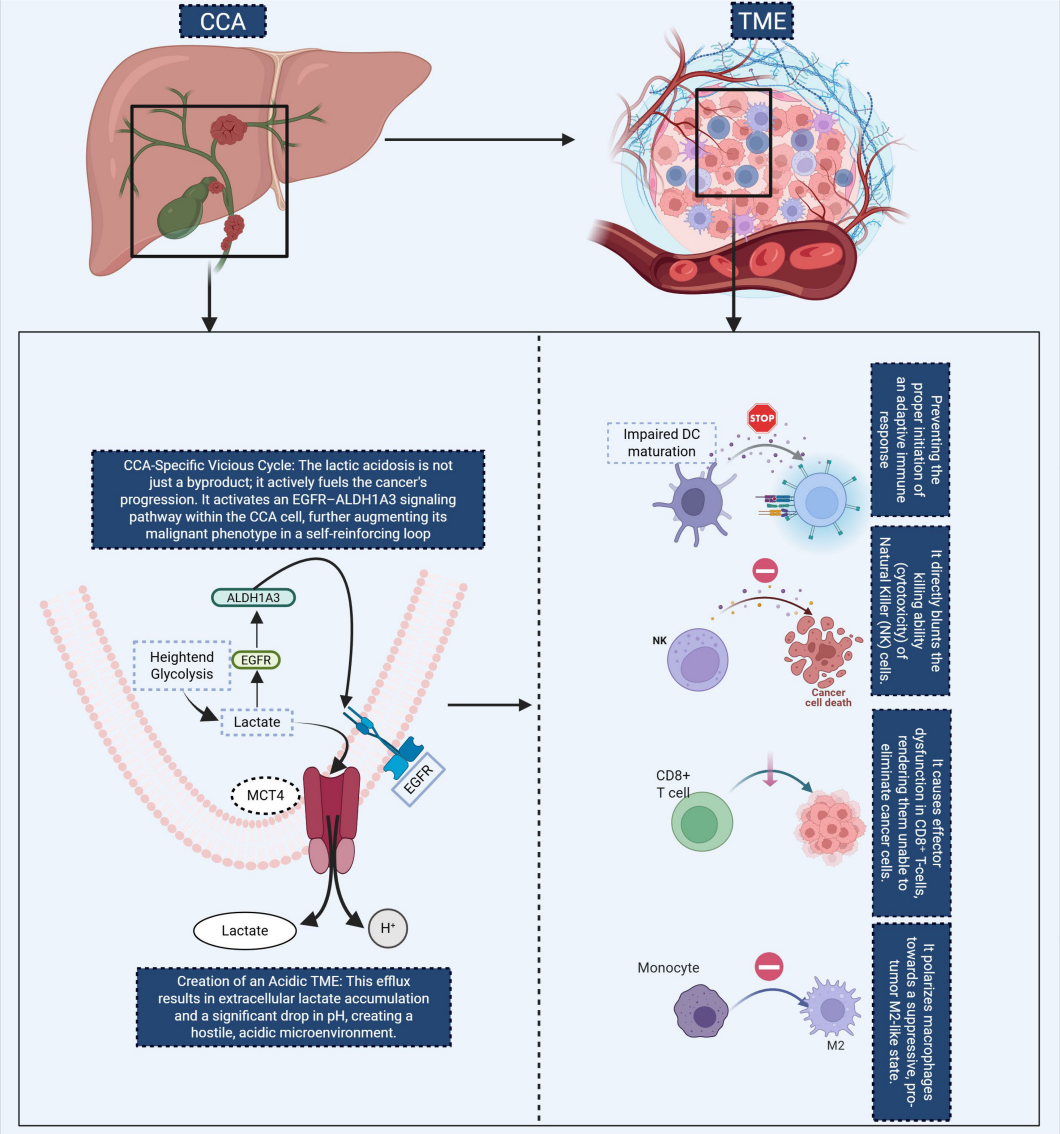
This diagram illustrates the self-reinforcing metabolic cycle in CCA and its effects on the TME. In CCA cells, lactate accumulation from heightened glycolysis activates the EGFR-ALDH1A3 signaling pathway, further promoting glycolysis and contributing to the malignant phenotype. This results in lactate efflux via MCT4, creating an acidic TME that impairs immune function. The figure highlights key immune alterations in the TME, including impaired DC maturation, which inhibits the activation of adaptive immune responses, and direct suppression of NK cell cytotoxicity, reducing antitumor immunity. Additionally, CD8+ T cell function is impaired, and monocytes are skewed toward an immunosuppressive M2 macrophage phenotype, further exacerbating immune evasion. These metabolic and immune alterations promote cancer progression by both directly fueling tumor growth and preventing effective immune surveillance. CCA, Cholangiocarcinoma; TME, tumor microenvironment; DC, Dendritic Cell; NK, Natural Killer.

Mechanistically, tumors with high lactate output and MCT4-dependent efflux polarize macrophages toward suppressive states and limit CD8^+^ infiltration (38); converging evidence across cancers indicates that therapeutic interference with lactate production/transport (e.g., LDH/MCT inhibition) can convert non-inflamed microenvironments to T-cell–permissive states (39) (**Figure 2**).

### 3.2 Amino-acid metabolism: tryptophan–kynurenine–AhR, arginine deprivation, and glutamine addiction

**Figure 3:**
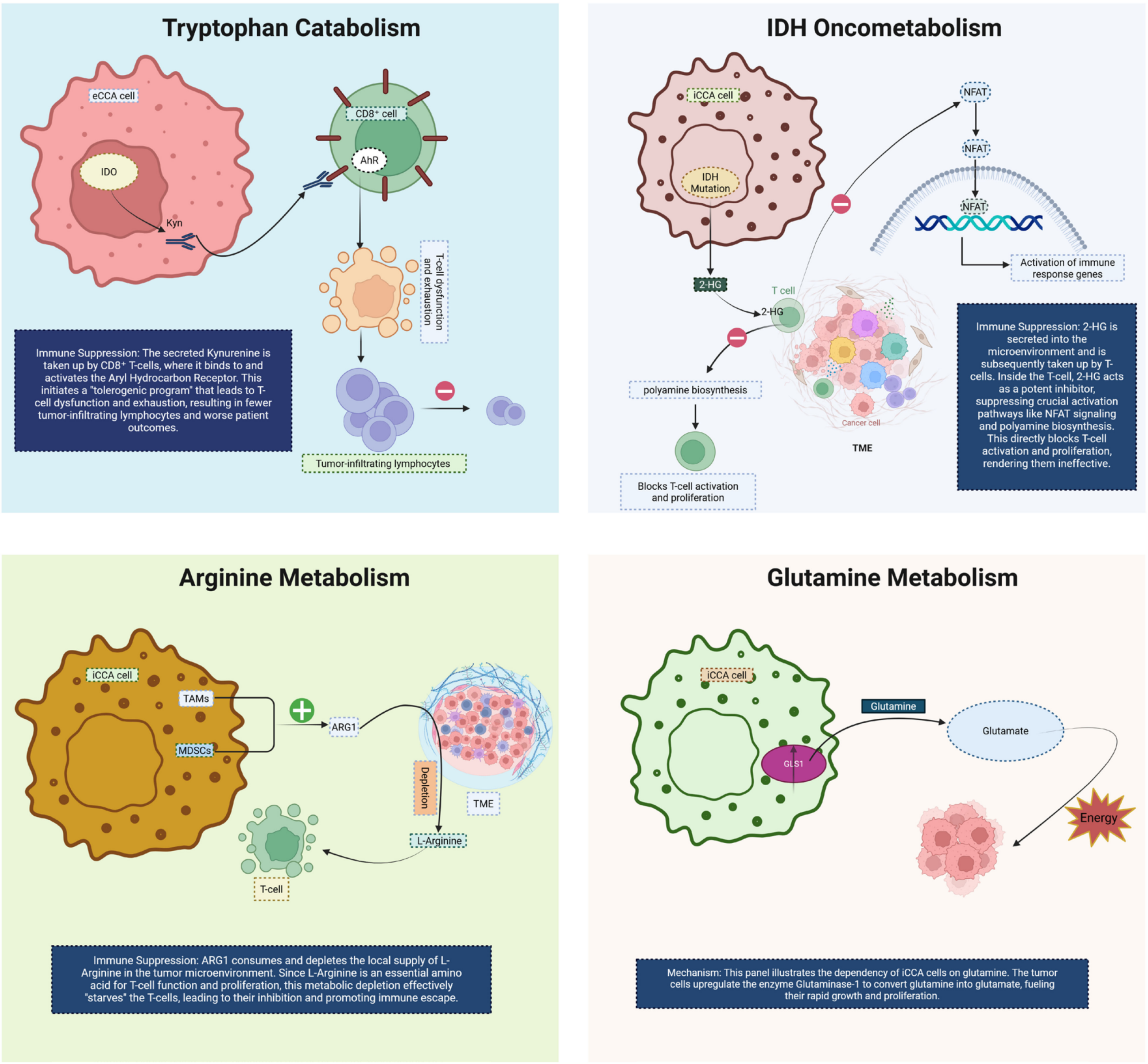
This diagram illustrates key metabolic pathways in CCA and their role in immune suppression and tumor progression. In the top-left panel, tryptophan catabolism via IDO leads to the production of kynurenine, which is taken up by CD8+ T cells. Binding of kynurenine to the AhR induces T cell exhaustion and dysfunction, resulting in fewer tumor-infiltrating lymphocytes and poorer patient outcomes. The top-right panel highlights IDH oncometabolism, where IDH mutations in CCA cells produce 2HG, which inhibits T cell activation by altering NFAT signaling, polyamine biosynthesis, and T cell proliferation, thus impairing immune responses. In the bottom-left panel, arginine metabolism is altered by TAMs and mesenchymal stem cells, which upregulate ARG1 expression. This leads to local depletion of L-arginine, impairing T cell activation and proliferation, thereby promoting immune evasion. Finally, the bottom-right panel shows glutamine metabolism in CCA cells. Tumor cells upregulate the enzyme glutaminase, converting glutamine to glutamate, which provides energy for rapid growth and proliferation. Together, these metabolic changes facilitate immune escape and tumor progression in CCA. CCA, Cholangiocarcinoma; eCCA, extrahepatic Cholangiocarcinoma; iCCA, Intrahepatic cholangiocarcinoma; IDO, Indoleamine 2,3-dioxygenase; AhR, Hydrocarbon receptor; 2-HG, R-2-hydroxyglutarate; TME, tumor microenvironment; TAMs, Tumor-associated macrophages; MDSCs, Myeloid-derived suppressor cells; ARG1, Arginase-1; GLS1, Glutaminase-1.

Recent evidence indicates that dysregulated amino-acid metabolism plays a pivotal role in immune evasion and tumour progression in cholangiocarcinoma (40). In extra-hepatic bile-duct carcinoma, elevated expression of indoleamine 2,3-dioxygenase 1 (IDO1) is significantly correlated with reduced infiltration of CD8^+^ tumour-infiltrating lymphocytes and poorer clinical outcomes, supporting the concept of a kynurenine–aryl-hydrocarbon-receptor (AhR) driven immunotolerant programme. In iCCA harbouring IDH1/2 mutations, the oncometabolite R-2-hydroxyglutarate (2-HG) can be taken up by T cells and has been shown to inhibit NFAT signalling and polyamine biosynthesis, thereby restricting T-cell activation and proliferation—an immunosuppressive mechanism that plausibly underlies poor ICI responsiveness in IDH-mutant iCCA (41). Furthermore, tumour-associated myeloid populations, including myeloid-derived suppressor cells (MDSCs) and tumour-associated macrophages (TAMs), overexpress arginase-1 (ARG1) and inducible nitric-oxide synthase (iNOS), depleting L-arginine and reshaping the microenvironment toward immune escape. On the glutamine axis, iCCA cells frequently overexpress glutaminase-1 (GLS1), and preclinical work (e.g., the GLS1 inhibitor CB-839) has shown that suppression of glutamine metabolism reduces malignant phenotypes and may sensitize tumours to immunotherapeutic intervention (42). These findings collectively underscore the importance of amino-acid metabolic pathways as both mechanistic drivers of immunosuppression and as actionable therapeutic nodes in cholangiocarcinoma (**Figure 3**).

### 3.3 Lipid programs and the eicosanoid pathway

**Figure 4:**
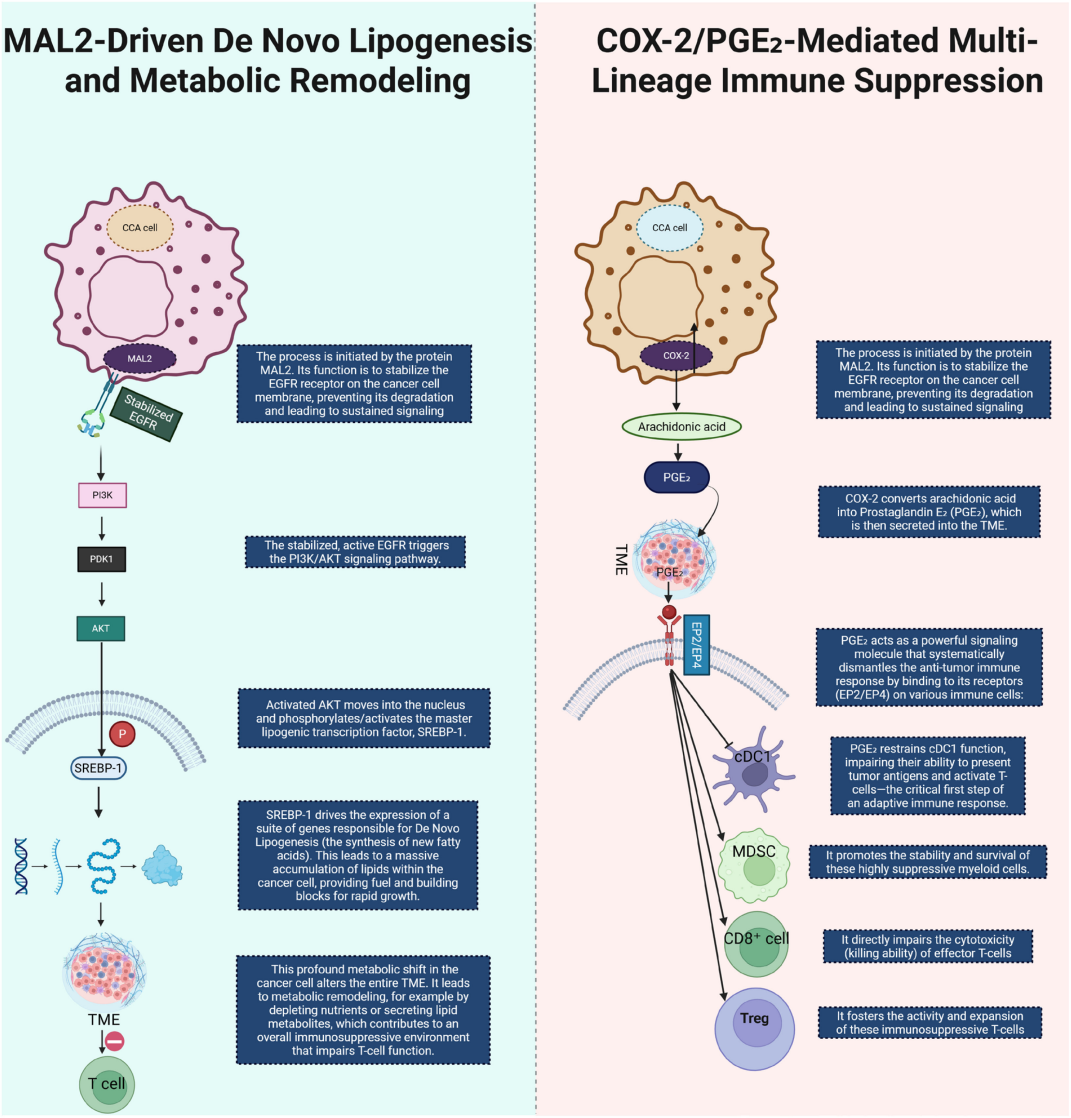
This diagram illustrates two key metabolic and immune-modulatory processes in CCA. On the left, MAL2-driven *de novo* lipogenesis is shown to initiate by stabilizing EGFR on the cancer cell membrane. This stabilization prevents EGFR degradation and leads to sustained EGFR signaling, triggering the PI3K/AKT signaling pathway. Activated AKT translocates to the nucleus, where it activates the lipogenic transcription factor SREBP-1. SREBP-1 induces the expression of genes responsible for lipid synthesis, promoting lipid accumulation within CCA cells, thus fueling tumor growth and contributing to an immune-suppressive TME that impairs T cell function. On the right, COX-2/PGE2-mediated immune suppression is depicted. COX-2 converts arachidonic acid into PGE2, which is secreted into the TME. PGE2 suppresses the immune response by binding to its receptors (EP2/EP4) on various immune cells. It inhibits the function of dendritic cells (DCs), promoting immune evasion, while enhancing the stability and expansion of MDSCs and Tregs, and impairing the cytotoxicity of CD8+ T cells. This multi-lineage immune suppression facilitates tumor immune evasion and progression in CCA. CCA, Cholangiocarcinoma; COX2, Cyclooxygenase-2; PGE_2_, Prostaglandin E_2_; TME, tumor microenvironment; Cdc1, Conventional Dendritic Cell, MDSC, Myeloid-Derived Suppressor Cell; Treg, Regulatory T-cell.

De novo lipogenesis (DNL) emerges as a key metabolic program in CCA. Recent single-cell and organoid analyses in iCCA identify MAL2 as a driver of lipid accumulation by stabilizing membrane EGFR localization and activating the PI3K/AKT/SREBP-1 axis—thus linking oncogenic signaling to lipogenic transcription and robust metabolic remodeling that shapes immune contexture (43). Elevated activity of SREBP-1 drives up-regulation of fatty acid synthase (FASN), stearoyl-CoA desaturase (SCD) and other DNL genes, correlating with increased phosphatidic acid levels, altered membrane composition and resistance to chemotherapy in iCCA models (44). Parallel to lipogenic enhancements, the prostanoid arm of the eicosanoid pathway—most prominently the PTGS2 (COX-2)/Prostaglandin E_2_ (PGE_2_) axis—plays a central role across tumor types including hepatobiliary cancers. PGE_2_ accumulates in the tumor microenvironment (TME), binds EP2/EP4 receptors and triggers downstream signaling (PKA, β-catenin, NF-κB, PI3K/AKT) that restrains conventional dendritic cell type 1 (cDC1) lineage function, enhances MDSC stability, suppresses CD8^+^ T-cell cytotoxicity and supports regulatory T-cell (Treg) activation (45). Importantly, pharmacologic inhibition of COX-2 or EP2/EP4 receptors has been shown to restore antigen presentation and cytotoxic responses in preclinical models—highlighting a metabolic lever to de-repress antitumor immunity in CCA. Together, these mechanistic insights position lipid programs and eicosanoid signaling at the heart of an immunometabolic barrier network that restricts effective immunotherapy, and suggest that targeting DNL or PGE_2_ pathways may convert immunologically “cold” CCA into more responsive states (**Figure 4**).

### 3.4 Purine metabolism and the adenosine axis

**Figure 5:**
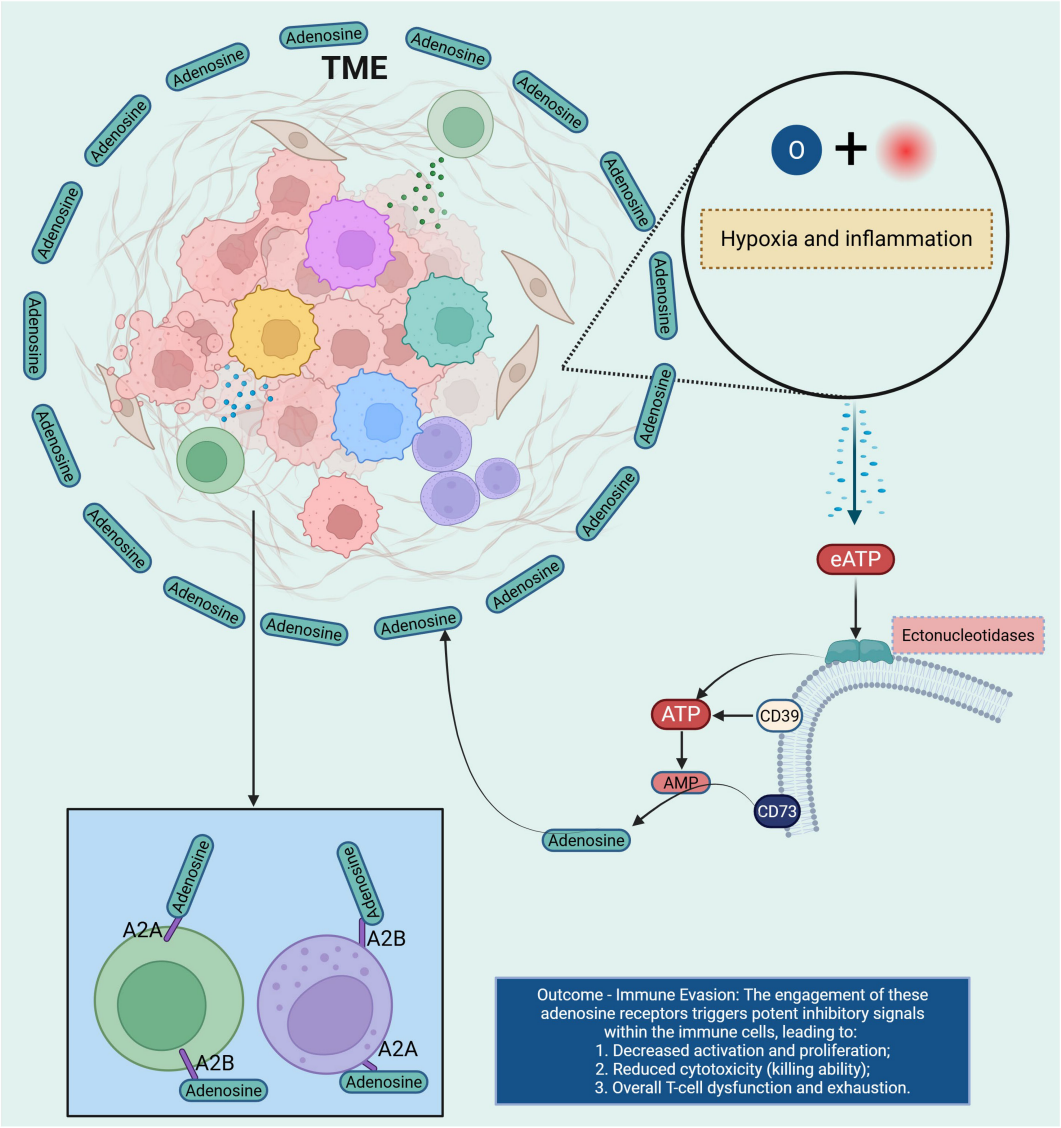
This diagram illustrates the role of adenosine receptors in immune suppression within the TME. Under conditions of hypoxia and inflammation, eATP is released into the TME. Ectonucleotidases, including CD39 and CD73, hydrolyze eATP into adenosine, which binds to adenosine receptors (A2A and A2B) on immune cells. The engagement of these receptors triggers potent inhibitory signals, leading to decreased activation and proliferation of immune cells, reduced cytotoxicity, and overall T cell dysfunction and exhaustion. This results in immune evasion, as adenosine signaling dampens the immune response, allowing the tumor to escape immune surveillance and progress. TME, tumor microenvironment; Extracellular ATP; AMP, Adenosine Monophosphate.

Purine metabolism plays a pivotal role in regulating immune responses within the TME. Adenosine, a key metabolite in purine catabolism, accumulates in the TME due to the elevated activity of ectonucleotidases such as CD39 and CD73; CD39 converts extracellular ATP/ADP to AMP, and CD73 further hydrolyzes AMP to adenosine (46, 47), thus shifting the balance from immunostimulatory ATP toward immunosuppressive adenosine. This accumulation leads to the engagement of adenosine receptors (A_1_R, A_2_AR, A_2_BR, A_3_R) on immune cells, thereby modulating immune functions: A_2_AR/A_2_BR activation in particular increases intracellular cAMP, inhibiting effector T-cell and NK-cell activity, promoting regulatory T-cells (Tregs) and MDSCs, and impairing dendritic cell (DC) antigen presentation. In the context of CCA, emerging evidence—though still limited—suggests that the adenosine axis contributes to immune evasion and tumor progression by creating a metabolically immunosuppressive niche. For example, hypoxia-driven upregulation of CD39/CD73 in hepatobiliary cancers correlates with increased adenosine production, reduced CD8^+^ T-cell infiltration, and worse survival outcomes. Pharmacologic blockade of CD39 or CD73, or antagonism of A_2_AR/A_2_BR, has been shown in preclinical models to restore antigen presentation, bolster CD8^+^ T-cell cytotoxicity, reduce Treg/MDSC abundance, and sensitize tumors to immune checkpoint inhibitors (ICIs) (48). Together, these mechanistic insights place the purine-adenosine axis at the center of an immunometabolic barrier network, indicating that targeting this pathway may convert immunologically “cold” CCA into more treatment-responsive states (**Figure 5**).

### 3.5 Bile-acid signaling (FXR/TGR5) at the metabolism–immunity interface

**Figure 6:**
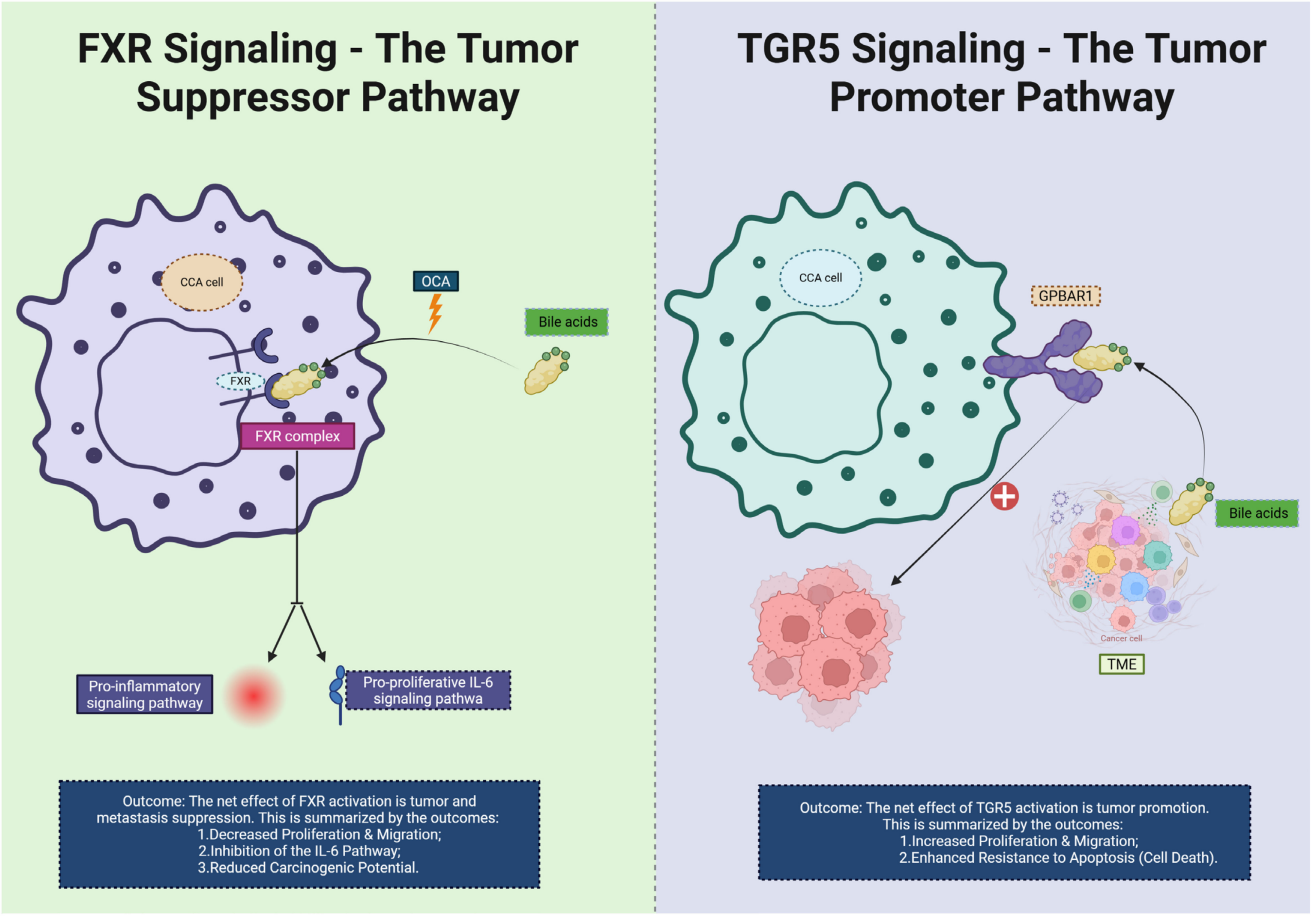
This diagram illustrates the contrasting roles of FXR and TGR5 signaling pathways in CCA. On the left, FXR signaling is shown as a tumor suppressor pathway. Bile acids activate FXR, which binds to the FXR complex in the CCA cell. This activation inhibits pro-inflammatory signaling and reduces the pro-proliferative IL-6 pathway, leading to decreased cell proliferation, migration, and a reduced carcinogenic potential. The net effect of FXR activation is the suppression of tumor progression and metastasis. On the right, TGR5 signaling acts as a tumor promoter. Bile acids activate TGR5 and upregulate the GPBAR1 receptor on the CCA cell, which stimulates increased cell proliferation, migration, and enhanced resistance to apoptosis (cell death). The overall effect of TGR5 activation is the promotion of tumor growth and immune evasion within the TME. CCA, Cholangiocarcinoma; TME, tumor microenvironment; OCA, Obeticholic Acid; FXR, Farnesoid X Receptor.

The biliary milieu indeed endows CCA with distinctive immunometabolic wiring. Recent studies show that FXR functions as a tumor/metastasis suppressor in intrahepatic CCA (iCCA), in part 1by inhibiting the IL-6/STAT3 signaling axis and epithelial-mesenchymal transition (EMT); activation of FXR by the agonist Obeticholic Acid (OCA) reduces CCA carcinogenic potential in preclinical models, dampens proliferative signaling (49, 50), and modifies the immune microenvironment by promoting dendritic cell antigen-presentation and reducing myeloid-derived suppressor cell (MDSC) infiltration. On the contrary, heightened expression of G-protein-coupled bile acid receptor (TGR5) in biliary epithelial cells and CCA specimens promotes cell proliferation, migration, resistance to apoptosis, and M2-like macrophage polarization via increased cAMP and ERK signaling. Furthermore, bile acids acting via FXR/TGR5 modulate immune-microenvironment cross-talk: for example, certain secondary BAs bind FXR in Kupffer cells to inhibit NF-κB activation and proinflammatory cytokine release (51, 52), while TGR5 activation in cholangiocytes triggers secretion of immunosuppressive cytokines (IL-10, TGF-β) and recruits regulatory T cells (Tregs). Emerging multi-omics analyses of the bile duct microenvironment in CCA reveal that alterations in BA pool composition (e.g., increased lithocholic acid, taurolithocholic acid) correlate with reduced CD8^+^ T-cell infiltration and higher checkpoint ligand (PD-L1) expression, suggesting that the BA–immune axis contributes to immune exclusion and therapy resistance. These mechanistic insights place bile-acid signaling squarely at the heart of an immunometabolic barrier network and highlight the translational promise of targeting the BA–FXR/TGR5 axis—either by restoring FXR activity, inhibiting TGR5, or modulating BA composition— as a strategy to convert immunologically “cold” CCA into more immune-responsive states (53). We believe these additions strengthen the section and enhance its relevance to current research in CCA immunometabolism (**Figure 6**).

### 3.6 Hypoxia/oxidative stress, mitochondrial programs, and checkpoint regulation

**Figure 7:**
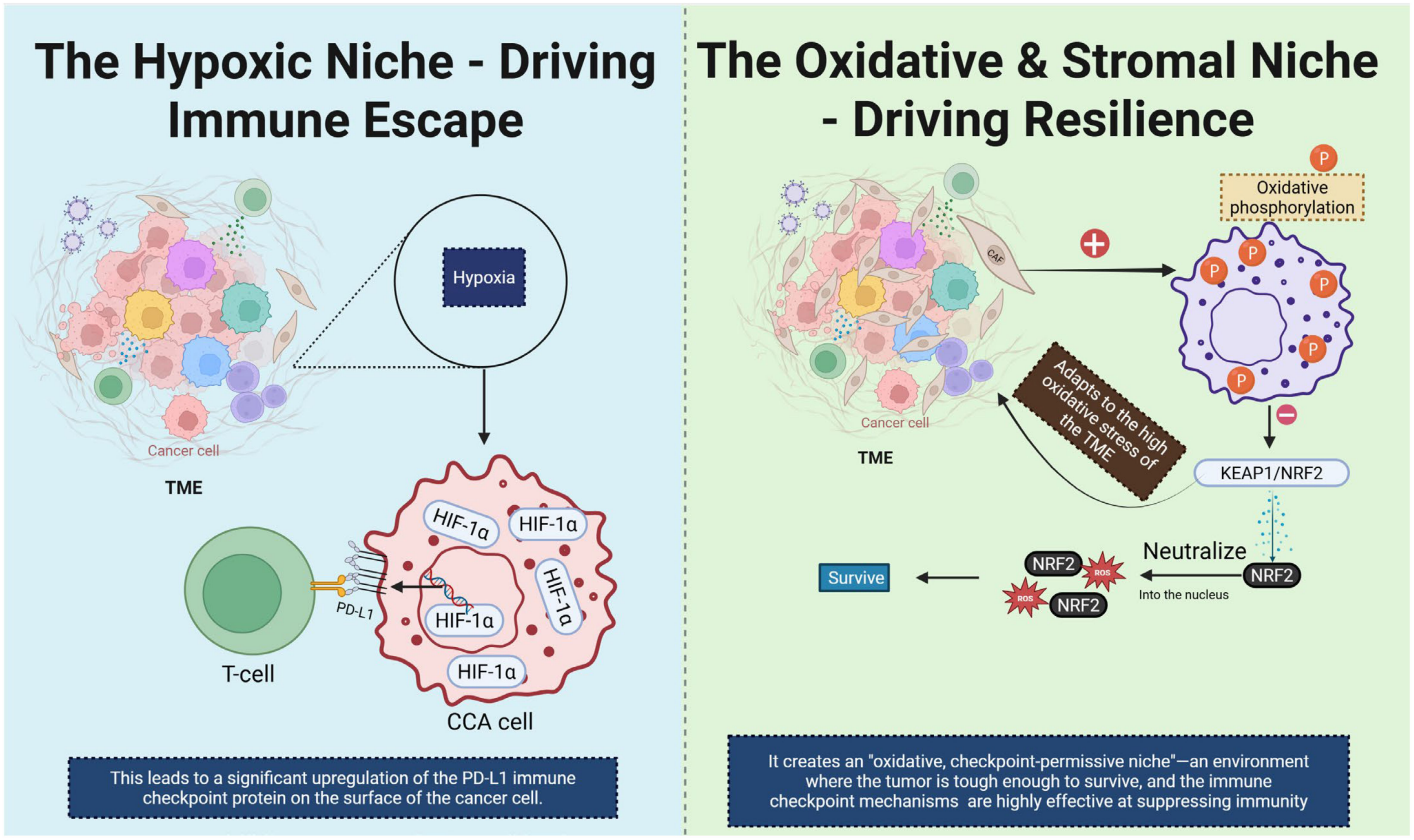
This diagram illustrates two distinct TME niches that contribute to immune escape and resilience in CCA. On the left, the hypoxic niche is depicted. Hypoxia in the TME leads to stabilization and activation of HIF-1α, which upregulates the expression of PD-L1 on CCA cells. This results in immune evasion by inhibiting T cell activation, as PD-L1 interacts with the PD-1 receptor on T cells. On the right, the oxidative and stromal niche is shown. High oxidative stress in the TME induces oxidative phosphorylation, activating the KEAP1/NRF2 signaling pathway. NRF2 translocates into the nucleus and promotes the expression of antioxidant genes, helping the tumor cells survive in the harsh oxidative environment. This creates an “oxidative, checkpoint-permissive niche” where the tumor becomes resilient to immune attacks and immune checkpoint mechanisms effectively suppress immune responses. CCA, Cholangiocarcinoma; TME, tumor microenvironment; CAF, Cancer-Associated fibroblast.

Hypoxia is a hallmark of the desmoplastic microenvironment in CCA and exerts profound effects on immunometabolism. Under low-oxygen conditions, HIF-1α is stabilized and translocates to the nucleus where it binds hypoxia-response elements (HREs) in the promoters of genes including PD-L1, thereby directly up-regulating immune-checkpoint expression and contributing to T-cell dysfunction and immune escape (54). Specific evidence in CCA shows that high HIF-1α expression correlates with advanced intrahepatic CCA (iCCA) progression, increased PD-L1 levels and diminished CD8^+^ T-cell infiltration (27). Parallel to hypoxia‐driven checkpoint regulation, mitochondrial and oxidative programs play a central role. In CCA models, enhanced mitochondrial OXPHOS supports cancer stem−cell features, e.g., increased sphere−forming capacity and resistance to therapy (55). Moreover, dysregulation of the KEAP1/NRF2 axis enables redox adaptation: NRF2 activation induces antioxidant genes, mitochondrial biogenesis and promotes survival under oxidative stress (56). Together these changes foster an oxidative, checkpoint-permissive niche where immune-effector cells are suppressed, while tumor cells exploit metabolic flexibility for growth and immune evasion. In the context of CCA, CAF-rich stroma further enhances these programs: fibroblast-secreted HGF and TGF-β create a microenvironment of reactive oxygen species (ROS) generation, promoting mitochondrial OXPHOS in tumor cells and reducing immune infiltration (57). Collectively, the convergence of hypoxia, mitochondrial remodeling, oxidative stress and checkpoint up-regulation defines a metabolic-immune barrier that undermines immunotherapy efficacy. Targeting this axis—via HIF-1α inhibitors, NRF2 pathway modulators or mitochondrial metabolism blockers—may re-sensitize CCA to immune-checkpoint inhibitors and shift the TME from suppressive to permissive (**Figure 7**).

## 4 Metabolic fates of immune and stromal cells in CCA

### 4.1 T cells: effector, memory, exhaustion, and regulatory lineages

Across BTCs—especially iCCA—T cells frequently reside in a metabolically hostile, myeloid-dominated stroma, where nutrient deprivation, acidity, and redox stress converge to impair activation and persistence. Exhausted CD8^+^ T cells exhibit reduced mitochondrial mass and fitness, depressed OXPHOS, and defective bioenergetic adaptability; genetic and pharmacologic studies demonstrate that mitochondrial insufficiency is not merely correlative but can drive the transcriptional and functional hallmarks of exhaustion (58, 59). Restoring mitochondrial biogenesis (e.g., by augmenting PGC-1α programs) improves cytotoxic function in chronic stimulation settings (60, 61), suggesting a lever to resuscitate anti-tumor responses in iCCA.

Regulatory T cells (Tregs) display a distinct metabolic wiring that favors lipid uptake and fatty-acid oxidation (FAO), supporting survival in glucose-poor, lactate-rich niches (62). In tumors, Treg expression of the fatty-acid transporter CD36 and lipid-handling programs (e.g., FABP5) sustains OXPHOS and suppressive capacity; genetic disruption of CD36 weakens intratumoral Treg stability and enhances effector T-cell activity. These features, together with broader evidence that T-cell subsets dynamically re-balance glycolysis and FAO to meet functional demands, underscore lipid metabolism as a manipulable axis to tip the effector–regulatory balance in CCA (63).

### 4.2 Myeloid cells: TAMs, MDSCs, and monocytes

Myeloid populations are prominent in iCCA and correlate with immune exclusion. TAMs accumulate lipids (including oxidized species), adopt an OXPHOS/FAO-skewed program, and polarize toward wound-healing (64), immunosuppressive states through PPAR/LXR signaling; blocking fatty-acid uptake (e.g., CD36) or FAO can recondition TAMs toward inflammatory phenotypes across tumor models (65, 66). Recent hepatobiliary data indicate that CCA cells can redirect fatty acids and reshape macrophage metabolism, reinforcing a lipid-fueled immunosuppressive loop relevant to the biliary niche (67).

MDSCs similarly rely on enhanced FAO and arachidonic-acid handling to sustain suppression (68), with FATP2-dependent uptake feeding PGE_2_ synthesis and CPT1-dependent FAO maintaining survival/function. Targeting these nodes diminishes MDSC activity and improves responses to immunotherapy in preclinical systems (69), supporting attempts to combine FAO/FATP2 blockade with checkpoint inhibitors in BTC.

Emerging evidence underscores that TAMs in the tumour microenvironment undergo profound metabolic reprogramming which drives their immunosuppressive and pro-tumour functions. For instance, lactate accumulation within hypoxic regions promotes TAM polarization toward an M2-like phenotype via histone and protein lactylation, thereby reinforcing immune suppression and tumour progression (70, 71). Concurrently, TAM lipid metabolism is altered, with increased fatty acid uptake and FAO associating with pro-tumour macrophage phenotypes; such lipid-metabolic shifts are particularly noted in liver cancers including CCA (72). Moreover, amino-acid metabolism and metabolic signalling (e.g., via arginine, tryptophan, and glutamine pathways) influence TAM functional states and thereby modulate anti-tumour immunity (73). Altogether, this evidence suggests that targeting TAM metabolic programmes—not merely generic macrophage depletion—offers a promising therapeutic strategy to reverse immune suppression and enhance efficacy of immunometabolic therapies in CCA and other tumours.

### 4.3 Dendritic cells (DCs) and NK cells: antigen presentation and cytotoxicity under metabolic stress

In cancer, lipid accumulation and lipid peroxidation in DCs impede peptide–MHC-I trafficking and block cross-presentation, thereby weakening CD8^+^ T-cell priming (74). Mechanistically, oxidatively truncated lipids packaged in lipid bodies are sufficient to disrupt cross-presentation (75), while excess neutral lipids from tumor-derived factors exert similar effects—findings that provide a biochemical rationale for lipid-normalizing strategies to restore DC function in iCCA.

Natural killer (NK) cells require both glycolysis and OXPHOS for degranulation and IFN-γ production; recent work shows FAO supports NK responses to tumors and viruses, whereas excessive uptake/accumulation of lipids (including oxidized species via CD36 or SR-B1) can blunt cytotoxicity (76, 77). These data argue that tuning fatty-acid flux—rather than simple fuel supplementation—may be necessary to preserve NK function in the lipid-rich biliary microenvironment (78).

### 4.4 B cells and plasmablasts: cholesterol/sterol control of humoral immunity

Humoral responses are metabolically gated. The sterol-regulation pathway, via SREBP signalling, integrates intracellular cholesterol and sterol status to regulate B-cell activation and differentiation; notably, the cholesterol metabolite 25-hydroxycholesterol (25-HC) restrains SREBP2 and limits plasma-cell output in mucosal settings (79, 80). Activated B cells and plasmablasts increase mitochondrial OXPHOS and boost nutrient uptake (glucose, amino acids, lipids) to sustain high-rate antibody secretion; inhibition of mitochondrial ATP production curtails immunoglobulin titers, demonstrating dependence on mitochondrial bioenergetics (81). Beyond these mechanisms, recent work shows that sterol-metabolite-driven epigenetic reprogramming in B cells influences their fate: accumulation of oxysterols represses SREBP2, alters membrane lipid raft composition, and limits immune complex trafficking and plasmablast differentiation. Furthermore, in tumour or inflamed microenvironments, plasmablasts demonstrate enhanced OXPHOS together with increased mitochondrial mass and reactive oxygen species (ROS) signalling, linking metabolic stress with humoral dysregulation. These principles imply that in the unique biliary niche of intrahepatic CCA (iCCA) — characterised by altered sterol handling, cholestatic stress, and mitochondrial-stress conditions — B-cell function and tertiary lymphoid structure formation may be skewed. Specifically, disrupted cholesterol/sterol metabolism could impair antigen-driven B-cell differentiation, reduce high-affinity antibody production, or favour immunosuppressive B-cell subsets, thereby impacting local humoral immunity and the immuno-metabolic axis in CCA (82).

### 4.5 CAFs and endothelial cells: nutrient routing, matrix cues, and barrier functions

CCA is profoundly desmoplastic. Fibroblast-derived LOX—abundant in iCCA stroma—increases tumor OXPHOS and stemness, linking matrix cross-linking to metabolic hard-wiring and aggressive behavior; pharmacologic LOX inhibition remodels the fibro-inflammatory stroma and sensitizes CCA to therapy in preclinical studies (28). Moreover, bile acids can activate CAFs and drive an immunosuppressive microenvironment in CCA (33), highlighting a disease-specific stromal axis upstream of immune dysfunction (83).

Beyond matrix cues, CAFs and tumor endothelium participate in metabolite shuttles that shape local immunity. Lactate and adenosine are emblematic: CAFs and endothelial cells express ecto-nucleotidases and can contribute to adenosine production, while endothelial MCT1-mediated lactate influx triggers NF-κB/IL-8 signaling and pro-angiogenic programs that indirectly enforce immune exclusion. Together with iCCA-intrinsic upregulation of CD73 (84), these circuits suggest combined strategies that target stromal transport/enzymes (MCTs, CD73) alongside immune checkpoints (39, 48).

Recent investigations highlight that CAFs in CCA are highly heterogeneous—comprising multiple sub-types with both tumour-promoting and tumour-restricting functions—and actively engage in extensive crosstalk with tumour cells, immune infiltrates and extracellular matrix (ECM) via metabolic, signalling and biomechanical pathways (29). Mechanistically, CAFs remodel ECM to create physical and biochemical barriers to drug delivery and immune infiltration, secrete cytokines and growth factors (such as IL-6, SDF-1, periostin) that foster immunosuppression and angiogenesis, and undergo metabolic reprogramming themselves—enhanced glycolysis, increased glutamine or lipid utilisation—to sustain tumour growth and modulate the immune-metabolic microenvironment (85). From a therapeutic standpoint, this rewiring underscores why simple depletion of CAFs (or wholesale matrix destruction) may be insufficient or even harmful—past strategies in other desmoplastic tumours (e.g., pancreatic cancer) have shown paradoxical acceleration of disease when CAFs are indiscriminately ablated. Instead, recent evidence calls for approaches that reprogram CAF subsets from immunosuppressive to immunostimulatory phenotypes, inhibit specific tumour-stromal metabolic circuits, or target the tumour-CAFs cross-talk axis to restore immune access and drug delivery (86). Overall, integrating stroma-targeted strategies within immuno-metabolic frameworks may be indispensable for overcoming the resistant “cold” phenotype of CCA and improving therapeutic efficacy.

## 5 Immune checkpoints and metabolic cross-talk in CCA

### 5.1 PD-1/PD-L1 and CTLA-4 with metabolic reprogramming

In CCA, the immune checkpoint pathways PD-1/PD-L1 and CTLA-4 play pivotal roles in immune evasion (87–89). The binding of PD-1 on T cells to PD-L1 on tumor cells or stromal cells leads to T-cell exhaustion and immune suppression. Metabolic reprogramming within the TME influences the expression of PD-L1, creating a feedback loop that perpetuates immune suppression. For example, hypoxia and lactate accumulation in the TME induce HIF-1α stabilization, which in turn upregulates PD-L1 expression, thereby inhibiting T-cell-mediated cytotoxicity (90). Additionally, PD-1/PD-L1 interaction promotes a metabolic shift towards glycolysis and reduced mitochondrial OXPHOS in effector T cells, thereby limiting their function. Inhibiting this pathway has shown promise in other cancers (91), and combination therapies targeting both PD-1/PD-L1 and metabolic pathways (e.g., lactate dehydrogenase [LDH] inhibitors or metabolic reprogramming agents) are being investigated to improve treatment efficacy in CCA (34, 92).

Similarly, CTLA-4 is a critical immune checkpoint that regulates T-cell activation by binding to CD80/86 on antigen-presenting cells (APCs), leading to downregulation of T-cell responses. Recent findings have suggested that metabolic reprogramming in CCA influences CTLA-4 expression (93). For instance, glucose deprivation in tumors promotes the activation of AMPK, which regulates mTORC1 activity and CTLA-4 expression, ultimately impairing T-cell activation and promoting immune tolerance. Combination therapies targeting CTLA-4 and metabolic checkpoints such as mTOR inhibitors have shown potential in enhancing anti-tumor immunity and may offer a more effective therapeutic approach for CCA patients (94).

### 5.2 Secondary checkpoints (TIM-3, LAG-3, TIGIT, VISTA, CD47–SIRPα) and metabolic consequences

In addition to PD-1/PD-L1 and CTLA-4, several secondary immune checkpoints, including TIM-3, LAG-3, TIGIT, VISTA (95), and CD47–SIRPα, play critical roles in immune evasion in CCA (96). These secondary checkpoints act in tandem with metabolic changes within the TME to promote immune suppression. For example, TIM-3 is upregulated on exhausted T cells and promotes immune tolerance in cancers, including CCA (97). It interacts with its ligand galectin-9 to inhibit T-cell function, especially in the context of lactate accumulation and acidosis, which are hallmarks of CCA (98). Similarly, LAG-3 interacts with MHC class II molecules and exerts an inhibitory effect on T-cell responses (99). The metabolic regulation of LAG-3 expression has been implicated in its role in immune tolerance; for instance, glutamine metabolism and AMPK activation can modulate LAG-3 levels, further dampening immune responses in CCA (100).

TIGIT is another emerging checkpoint that inhibits T-cell activation by binding to CD155 and CD112 on tumor cells and APCs (101). TIGIT expression is influenced by the tumor metabolic microenvironment, with glucose deprivation and TGF-β signaling promoting its upregulation (102). The combination of TIGIT blockade and metabolic reprogramming has shown enhanced anti-tumor responses in preclinical models of solid tumors (89, 103), including CCA. Similarly, VISTA, a checkpoint that suppresses T-cell function, is also regulated by metabolic stress (104). Research has shown that VISTA expression is increased under conditions of hypoxia and lactate accumulation (105), which further exacerbates immune escape in CCA. Targeting VISTA in combination with metabolic inhibitors holds promise for improving immune responses in CCA patients (106).

Another critical immune checkpoint, CD47–SIRPα, acts as a “don’t eat me” signal to macrophages, preventing the phagocytosis of tumor cells (107). In CCA, high expression of CD47 correlates with poor prognosis and immune evasion (108). This pathway is regulated by metabolic cues, such as ROS and lipid metabolism, which induce CD47 expression in tumor cells (109). Targeting CD47–SIRPα in combination with metabolic inhibitors may enhance the phagocytosis of tumor cells and improve the efficacy of immunotherapies in CCA (110).

### 5.3 Metabolic pathways as therapeutic targets in ICB

The interplay between metabolic reprogramming and immune checkpoint signaling in CCA provides a rationale for combining metabolic inhibitors with ICB therapies (111). Recent clinical and preclinical studies have highlighted the therapeutic potential of targeting lactate production (via LDH inhibition), glutamine metabolism (via GLS inhibitors), and FAO (via CPT1 inhibitors) to enhance T-cell responses and overcome immune evasion (112). These metabolic inhibitors can directly impact immune cell function and shift the balance between effector and regulatory immune cells in the TME (113).

Combination therapies that target immune checkpoints and metabolic pathways are under investigation, with promising results observed in other cancers (114). For example, preclinical studies combining anti-PD-1 and glutamine metabolism inhibitors have demonstrated improved anti-tumor immunity and survival in mouse models of lung cancer and melanoma (115), and similar strategies may be applicable to CCA. Additionally, clinical trials exploring the combination of metabolic modulators with ICIs are underway, with the hope of enhancing therapeutic responses and overcoming resistance in CCA patients (116). These combinations aim to reprogram the immune microenvironment by both inhibiting the metabolic pathways that fuel tumor immune evasion and simultaneously restoring effective T-cell-mediated anti-tumor immunity.

### 5.4 The interaction between tumor metabolism and systemic metabolism and its clinical implications

Recent evidence indicates that tumour‐intrinsic metabolic reprogramming drives systemic metabolic perturbations, linking local tumour behaviour to whole‐body metabolic decline. Cancer cachexia—a syndrome characterised by skeletal muscle wasting, fat loss and elevated energy expenditure—is increasingly recognised as a consequence of tumour‐host metabolic crosstalk (113, 117). In patients with hepatic malignancies including CCA, metabolic disorders such as insulin resistance, non-alcoholic fatty liver disease and adipose dysfunction frequently co-exist and may predispose to poorer outcomes, suggesting a bidirectional interface between tumour metabolism and host systemic metabolic status (118). Mechanistically, tumours may secrete lipolytic and proteolytic factors, stimulate FAO, and promote adipose tissue browning and skeletal‐muscle catabolism, thereby sustaining their own nutrient supply while inducing host catabolism and reduced treatment tolerance (119). Taken together, these findings highlight the need to consider not only the intratumoral metabolic phenotype but also the systemic metabolic state when designing precision therapies—especially in BTCs where liver and metabolism are intimately connected.

### 5.5 Future perspectives and challenges

While the combination of metabolic inhibitors and ICB holds great promise for improving treatment outcomes in CCA, several challenges remain (120). The metabolic heterogeneity of CCA, along with the complex interactions between tumor cells, stromal cells, and immune cells, complicates the development of universally effective treatments (121). Additionally, tumor resistance to both ICIs and metabolic therapies, due to compensatory metabolic pathways or immune checkpoint redundancy, remains a significant hurdle (122). Future research should focus on the identification of specific metabolic vulnerabilities in CCA that can be targeted synergistically with immunotherapies (123), as well as the development of personalized treatment strategies based on individual tumor metabolic profiles. Further clinical trials and mechanistic studies will be essential to optimize combination strategies and overcome resistance mechanisms (124).

## 6 Spatial and temporal heterogeneity: from single-cell to spatial omics

### 6.1 Emergence of spatial transcriptomics and technological advancements

Spatial transcriptomics (ST) has revolutionized our understanding of tissue architecture by enabling the mapping of gene expression to specific tissue locations (125, 126). Unlike traditional bulk RNA sequencing, ST preserves the spatial context of gene expression, allowing for the examination of cellular heterogeneity within tissues (127). This technology integrates tissue sectioning with high-throughput RNA sequencing, providing a comprehensive view of the transcriptomic landscape *in situ*. The development of various ST platforms, such as Slide-seq, 10x Genomics Visium, and DBiT-seq, has expanded the resolution and applicability of ST, facilitating its use in diverse research areas, including cancer biology, neuroscience, and developmental biology.

### 6.2 Spatial heterogeneity in CCA

CCA, a malignancy originating from the bile ducts, exhibits significant spatial heterogeneity that influences tumor progression and response to therapy (128). Studies employing ST have delineated distinct TMEs within CCA, revealing variations in cellular composition and gene expression across different tumor regions (129). For instance, research by Zuyin et al. (2025) demonstrated that tumor cells at the leading-edge area of iCCA exhibit enhanced proliferation and are closely associated with stromal components, including endothelial cells and POSTN+ FAP+ fibroblasts. These interactions contribute to the formation of a unique “triad structure” that fosters tumor growth and progression (125).

Furthermore, Dong et al. utilized spatially resolved transcriptomics to investigate the cross-talk between cancer cells and TAMs in iCCA. Their findings indicated that tumor subclones are primarily driven by physical proximity within the TME, with macrophages playing a pivotal role in modulating the immune landscape and promoting tumor progression (128).

### 6.3 Integration of single-cell and spatial omics

The combination of single-cell RNA sequencing (scRNA-seq) and ST provides a powerful approach to unravel the complexities of the TME (130). scRNA-seq offers insights into the gene expression profiles of individual cells (131), while ST maps these profiles to their respective locations within tissue sections (132). Integrating these technologies allows for the identification of spatially distinct cell populations and the elucidation of their interactions within the TME (133).

**Figure 8:**
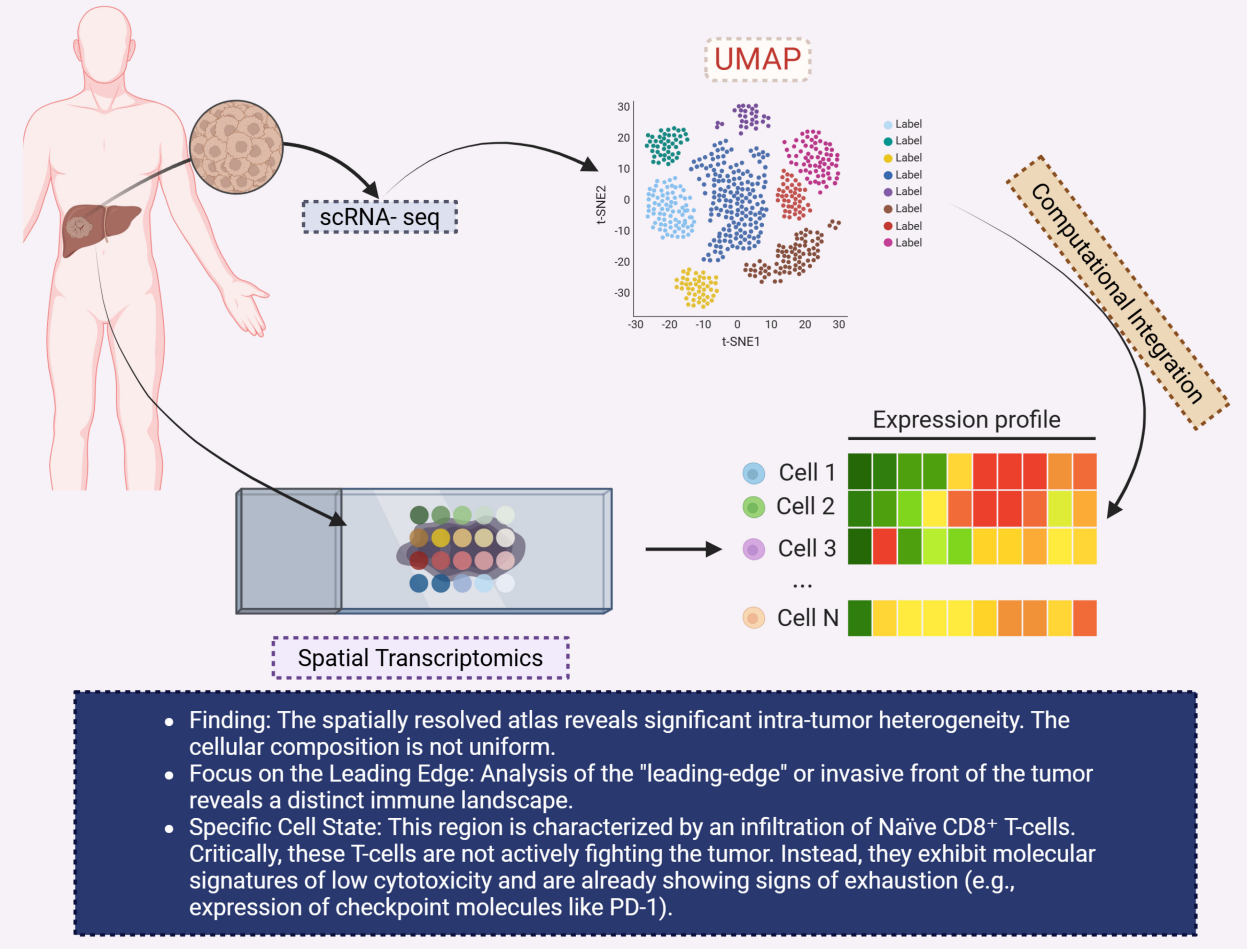
This diagram highlights the application of spatial transcriptomics and single-cell RNA sequencing (scRNA-seq) to investigate intra-tumor heterogeneity in CCA. The left section shows the tumor sample within the liver, which is analyzed using scRNA-seq to generate spatially resolved expression profiles. A UMAP plot (top right) visualizes the clustering of different cell types within the tumor, based on gene expression. The expression profiles of individual cells (bottom right) are shown in a heatmap, with varying expression levels of key genes across different cell populations (Cell 1 to Cell N). The findings indicate significant intra-tumor heterogeneity, particularly in the “leading-edge” or invasive front of the tumor, which is characterized by an infiltration of naïve CD8+ T-cells. These T-cells, however, are not actively fighting the tumor but exhibit molecular signatures of low cytotoxicity and early signs of exhaustion, such as the upregulation of immune checkpoint molecules like PD-1. The use of computational integration enables a deeper understanding of the immune landscape within CCA. CCA, Cholangiocarcinoma; UMAP, Uniform manifold approximation and projection.

In the context of CCA, this integrated approach has revealed heterogeneity in immune cell infiltration and stromal composition across different tumor regions. For example, Li et al. identified distinct immune landscapes in the leading-edge area of iCCA, characterized by the presence of naïve CD8+ T cells exhibiting low cytotoxicity and signs of exhaustion (134). These observations underscore the importance of spatial context in understanding immune evasion mechanisms in CCA (**Figure 8**).

### 6.4 Considerations for sample size, selection criteria, and statistical endpoints in multi-omics and spatial omics studies

With the widespread application of multi-omics and spatial omics technologies in cancer research, the importance of sample size, selection criteria, and statistical endpoints in study design has become increasingly evident. Recent guidelines suggest that each group (e.g., tumor subtypes) should include at least 26 samples to enhance group differentiation, while feature selection should be limited to no more than 10% of the total omics features, ensuring balanced sample proportions between categories, ideally not exceeding a 3:1 ratio, to improve analysis stability and reproducibility (135). Additionally, multi-omics integrative studies often face the “small n, large p” dimensionality disaster, where feature selection, sample heterogeneity, missing data, and platform differences impact statistical endpoint settings (e.g., survival, response rates, and the power required for subtype stratification) (136). For example, in spatial multi-omics studies, there is currently no standardization for sample acquisition, field of view (FOV), or replication numbers, which limits cross-study comparability and translational potential (137). Therefore, to ensure scientific rigor and generalizability, it is recommended that study designs: 1) calculate sample sizes based on expected effect sizes and subtype stratification needs; 2) include selection criteria encompassing tissue/liquid sources, processing workflows, and patient characteristics; 3) clearly define statistical endpoints (e.g., OS, PFS, biomarker-response rates) and incorporate pre-specified power analyses. This approach will ensure that multi-omics/spatial omics studies generate not only high-dimensional data but also reliable biomedical value and clinical translation potential.

### 6.5 Spatial omics in cholangiocarcinoma: integration of metabolic and immune profiling

Several recent studies have applied spatial omics technologies, such as ST and digital spatial profiling (DSP), to examine the spatial distribution of immune and metabolic features in iCCA. For example, one study found that tumor cell subclones are spatially distributed in close association with the TME, particularly at the tumor-stroma interface where TAMs are enriched (128). This region is characterized by low immune infiltration, activation of hypoxia-inducible factor 1 (HIF-1) signaling, and metabolic stress, highlighting the spatial overlap between immune suppression and metabolic challenges within the TME. Another review on liver and biliary tumors highlighted how spatial omics can visualize metabolic niches associated with immune exclusion. This study suggests that immune cell populations, particularly TAMs, are localized in areas with high metabolic stress, such as low oxygen and nutrient supply zones, leading to immune suppression (138). Additionally, while direct spatial omics data for iCCA are limited, there are emerging studies from related tumors, like pancreatic cancer, where the spatial co-localization of metabolic and immune factors is becoming increasingly apparent (139). These findings underscore the feasibility of using spatial omics to integrate metabolic reprogramming with immune profiles, offering a novel approach for understanding the spatial dynamics of immune evasion and metabolic adaptation in iCCA.

### 6.6 Clinical implications and future directions

The application of spatial and single-cell omics in CCA research holds significant promise for advancing personalized medicine. By providing detailed maps of the TME, these technologies can inform the development of targeted therapies aimed at specific tumor regions or cellular subsets. Moreover, the identification of spatially distinct biomarkers may facilitate the stratification of patients based on their tumor’s molecular and immune characteristics, leading to more tailored and effective treatment strategies (140).

However, challenges remain in translating these findings into clinical practice. The high cost and technical complexity of spatial omics platforms limit their widespread adoption. Additionally, the integration of spatial data with other omics layers, such as proteomics and metabolomics, requires further refinement to fully capture the multifaceted nature of the TME (141).

Future research should focus on optimizing spatial omics technologies to enhance their resolution and throughput, as well as developing computational tools to integrate and analyze multi-omics data. Collaborative efforts between researchers, clinicians, and technology developers will be essential to overcome existing barriers and realize the potential of spatial and single-cell omics in improving outcomes for CCA patients (142).

## 7 Therapeutic strategies: unlocking immunotherapy through metabolic reprogramming

### 7.1 Metabolic pathway inhibitors as ICI sensitizers

#### 7.1.1 IDO/TDO-AhR Axis inhibition as a new therapeutic strategy

The IDO/TDO-AhR axis plays a critical role in immune evasion and metabolic reprogramming in many cancers, including CCA. Both IDO and tryptophan 2,3-dioxygenase (TDO) catalyze the degradation of tryptophan into kynurenine (Kyn), which activates the aryl hydrocarbon receptor (AhR) on immune cells, leading to immune suppression through mechanisms such as reduced T-cell and NK-cell activity, increased regulatory T-cell (Treg) and myeloid-derived suppressor cell (MDSC) infiltration, and altered antigen presentation (143). Recent studies have highlighted novel small-molecule inhibitors targeting the IDO/TDO-AhR axis that show promise as sensitizers for ICIs in preclinical models of various cancers, including CCA (144). These inhibitors reduce Kyn levels, enhance T-cell responses, and reverse the immunosuppressive TME. For example, dual inhibition of IDO1 and TDO2 has shown to reduce Kyn accumulation and restore effector T-cell function, providing a rationale for combination therapies in CCA. However, challenges remain, including compensatory upregulation of TDO2 and alternative tryptophan-catabolic pathways that limit single-agent efficacy. Recent clinical trials, such as the ECHO-206 trial, combining IDO1 inhibitors with ICIs, have demonstrated modest success, underscoring the need for combination approaches (145). Given the immune-suppressive, hypoxic, and desmoplastic environment in CCA, targeting the IDO/TDO-AhR axis, along with metabolic and ICB, may offer new avenues for enhancing anti-tumor immunity in CCA patients. Future studies should focus on biomarker identification and patient stratification to optimize therapeutic outcomes.

#### 7.1.2 Adenosine pathway (CD39/CD73/A2A/A2B)

The adenosine pathway—mediated by CD39, CD73, and A_2_A/A_2_B receptors—is increasingly recognized as a major immunoregulatory checkpoint within the TME. CD39 (ENTPD1) and CD73 (NT5E) sequentially convert extracellular ATP/ADP to AMP and then to adenosine (ADO), resulting in accumulation of ADO in the TME which suppresses antitumor immunity (146). Elevated expression of CD39 and CD73 is found not only on tumor-associated immune cells such as regulatory T cells (Tregs), MDSCs, and macrophages, but also on malignant cells and CAFs. Preclinical studies demonstrate that high CD73 expression correlates with a “cold” immune microenvironment, reduced CD8^+^ T-cell infiltration, and poor outcomes in intrahepatic cholangiocarcinoma (iCCA). Pharmacologic inhibition of CD73 or blockade of A_2_A/A_2_B receptors has been shown to decrease extracellular adenosine levels (147), restore CD8^+^ T-cell and NK-cell cytotoxic activity, reduce Treg/MDSC populations, and enhance the efficacy of immune-checkpoint inhibitor (ICI) therapy in murine models. For instance, dual A_2_A/A_2_B receptor antagonist M1069 significantly reduced tumor growth *in vivo* and abrogated adenosine-mediated immune suppression. Moreover, recent investigations in iCCA show that ICI therapy itself can up-regulate CD73 expression via TNF-α/NF-κB signalling, suggesting adaptive resistance mechanisms which bolster the rationale for combining adenosine-axis inhibitors with PD-1/PD-L1 blockade. Collectively, these findings indicate that targeting CD39/CD73/A_2_A/A_2_B represents a promising strategy to overcome immune-exclusion in CCA and convert immunologically “cold” tumors into “hot,” treatment-responsive states (84).

#### 7.1.3 Lactate/MCT1/4, carbonic anhydrases, pH regulation

The TME of CCA is frequently characterized by extracellular acidification, a direct consequence of high rates of glycolysis in tumor cells and subsequent lactate efflux. Monocarboxylate transporters MCT1 and MCT4 mediate lactate and proton export across the plasma membrane, thereby sustaining intracellular pH homeostasis while acidifying the extracellular milieu (39, 148). Elevated expression of MCT1/4 is correlated with poor prognosis, increased metastatic potential, and immune evasion across cancers. Recent reviews demonstrate that lactate accumulation drives immunosuppressive effects via multiple mechanisms: suppression of CD8^+^ T-cell cytotoxicity, skewing macrophages toward M2 phenotypes, and promoting histone lactylation, which reprograms both tumor and immune-cell gene expression. In parallel, carbonic anhydrases (CAs), particularly CA IX and CA XII, catalyze hydration of CO_2_ to bicarbonate and protons, contributing further to extracellular acidosis. In hepatobiliary and other cancers, CA IX overexpression is tied to hypoxia, stromal activation, and a low-pH niche that impairs effector immune infiltration. Therapeutic inhibition of MCT1/4 (e.g., with AZD3965) or CAs has been shown in preclinical models to raise extracellular pH, reduce lactate-mediated immunosuppression, restore CD8^+^ T-cell and NK-cell function, and enhance responsiveness to ICIs (149). Although direct data in CCA remain limited, the mechanistic parallels strongly suggest that targeting lactate export and pH regulators may convert “immune-cold,” acidified CCAs into more immunotherapy-sensitive phenotypes. Together, the intersection of lactate transport and pH regulation via MCTs and CAs presents a compelling axis at the metabolism–immunity interface, meriting further exploration in CCA translational research (150).

#### 7.1.4 Glutamine metabolism inhibition (GLS, FAO Inhibition, FASN/ACC/SCD1 Inhibition)

Metabolic reprogramming in CCA involves glutamine metabolism, fatty acid oxidation (FAO), and lipid synthesis—all of which contribute to tumor cell survival and immune evasion. Recent work demonstrates that GLS inhibitors (e.g., CB-839) reduce glutamine metabolism, depriving tumor cells of key intermediates (glutamate, α-ketoglutarate) required for anabolic growth and redox balance (151). For example, in a rodent model of liver cancer, GLS inhibition decreased glutamine flux, lowered NADPH/ROS buffering capacity, and increased CD8^+^ T-cell infiltration and efficacy of ICIs. In parallel, FAO inhibitors such as etomoxir have been shown to impair fatty acid oxidation, reduce ATP supply and cancer stem-cell features, while FASN, ACC, and SCD1 inhibitors curb de novo lipid synthesis and desaturation, leading to altered membrane fluidity, reduced lipid signalling and enhanced immune recognition. In the specific context of CCA, a recent review on fatty acid metabolism in CCA highlights that CCA cells rely heavily on FAO and lipogenic enzyme overexpression (FASN, ACC) to support growth and immune escape (113). Combination therapies targeting GLS with FAO or lipid synthesis inhibitors plus ICI have demonstrated synergistic anti-tumor responses, including decreased Treg/MDSC proportions, enhanced effector T-cell cytotoxicity and improved survival in preclinical models. These combined strategies offer a promising approach to sensitizing CCA to immunotherapy by disrupting metabolic support for tumor cells and alleviating immunosuppression (152).

#### 7.1.5 Bile acid pathway (FXR/TGR5/FGFR4) and microbiome interventions

The bile acid signaling pathway, involving FXR, TGR5, and FGFR4, plays a crucial role in regulating inflammation, immune responses, and tumor progression in CCA (24). Activation of FXR and TGR5 can modulate immune cell infiltration and alter the TME. Recent studies have explored microbiome interventions and bile acid chelators as potential therapeutic strategies to modulate the TME and improve ICI efficacy in CCA. The gut microbiome’s role in shaping bile acid metabolism and its interaction with immune checkpoints in the liver suggests that microbiome-targeted therapies could enhance the effectiveness of ICIs in liver and biliary cancers (153).

Emerging data implicate the gut–liver (microbiome–bile acid) axis as a critical modulator of immuno-metabolic remodeling in CCA. Dysbiosis in CCA patients manifests as reduced microbial diversity, depletion of beneficial taxa (e.g., *Blautia* spp.) and enrichment of pathobionts, which correlates with altered bile acid pools and disrupted enterohepatic circulation (154, 155). Microbial transformations of primary bile acids into secondary bile acids (e.g., DCA, LCA) perturb host metabolic and immune signaling via receptors such as FXR and TGR5, thereby promoting tumor-friendly inflammation, immune-evasion (notably CD8^+^ T cell suppression) and fibrogenic/malignant progression (156). This mechanistic insight opens new therapeutic vistas: modulating the microbiome (via pre/pro-biotics, fecal microbiota transplant or targeted antibiotics) or bile acid metabolism may enhance immunotherapy efficacy and restore metabolic–immune homeostasis in CCA (157).

#### 7.1.6 Ferroptosis and lipid peroxidation induction

Ferroptosis, an iron-dependent form of regulated cell death characterized by lethal accumulation of lipid peroxidation products, has emerged as a promising therapeutic strategy in CCA/ (158). In CCA cells, aberrant iron metabolism and oxidative stress amplify peroxidation of polyunsaturated fatty acid-containing phospholipids, a process driven by the acyl-CoA synthetase long-chain family member 4 (ACSL4), and normally opposed by glutathione peroxidase 4 (GPX4). Recent studies in CCA confirm that induction of ferroptosis via lipid peroxidation—induced by agents such as erastin, RSL3 or other small-molecule inducers—reduces CCA cell proliferation and migration, and correlates with improved survival in preclinical models. Notably, one 2024 review highlights that CCA cells are highly sensitive to ferroptosis, and that ACSL4 and GPX4 are key regulators in CCA ferroptosis sensitivity (159). Moreover, emerging evidence links ferroptosis induction with enhanced anti-tumor immunity: lipid peroxidation in tumor cells leads to the release of immunogenic signals, increased tumor-infiltrating CD8^+^ T cells, and improved responses to immune checkpoint inhibitor (ICI) therapy in models of solid tumours. For example, inhibition of GPX4 in combination with anti-PD-1 therapy has been shown in other tumour types to promote ferroptosis and augment antitumour immune responses (160). Although specific combination data in CCA remain sparse, this mechanistic rationale strongly supports the integration of ferroptosis-inducing therapies (targeting ACSL4, GPX4 or other ferroptosis regulators) with ICIs in CCA (161). By triggering lipid peroxidation and overcoming metabolic and immune resistance pathways, these strategies may sensitize immunologically “cold” CCA tumours to checkpoint blockade, offering new avenues for translational and clinical research.

### 7.2 Tumor driver and metabolic co-targeting: FGFR, IDH1/2, PI3K-AKT-mTOR, MEK/ERK, YAP/TAZ and ICI/metabolic inhibitor triple therapy

Targeting tumor drivers such as FGFR, IDH1/2, PI3K-AKT-mTOR, MEK/ERK, and YAP/TAZ in combination with metabolic inhibitors offers a promising strategy to overcome resistance to ICIs (162). For instance, FGFR inhibitors in CCA (163), when used in combination with glutamine metabolism inhibitors, have demonstrated enhanced anti-tumor activity. The PI3K-AKT-mTOR pathway is frequently activated in CCA, promoting survival and proliferation (164). Inhibition of this pathway, combined with metabolic reprogramming agents (165), can lead to improved responses to ICIs. Preclinical studies have shown that targeting multiple pathways in a triple combination approach enhances efficacy and reduces immune evasion mechanisms (166).

In CCA, employing a “triplet” regimen that combines chemotherapy, ICIs and a metabolism-targeting agent holds promise for increasing efficacy but also raises substantial safety concerns. The liver-centric microenvironment of CCA—with frequent underlying hepatic dysfunction, cholestasis and bile-acid overload—renders patients especially vulnerable to off-target hepatotoxicity when fundamental metabolic pathways (for example fatty acid oxidation) are disrupted. Immune-related hepatotoxicity in ICI combinations is already well documented, ranging from mild transaminitis to fulminant hepatitis and cholangitis (167). Meanwhile, early phase studies in advanced BTCs suggest that triplet combinations may achieve acceptable safety profiles in selected populations; for instance, a recent trial in advanced BTC reported Grade 3–4 adverse events (AEs) comparable to doublet therapy (168). Nonetheless, the addition of a metabolism-targeting drug introduces two further layers of risk: first, inhibition of systemic or hepatic fatty acid metabolism may exacerbate liver injury or metabolic decompensation in patients with reduced hepatic reserve; second, overlapping toxicities (for example mitochondrial stress, oxidative injury, immune activation) may potentiate novel AEs. Accordingly, future trials of triplet regimens in CCA must incorporate rigorous hepatic safety monitoring (baseline liver function, bile duct injury biomarkers, dynamic metabolic panels), stratify patients by hepatic reserve and underlying biliary disease, and include prespecified limits and management algorithms for metabolic-pathway disruptions. Only by proactively addressing these safety challenges can the enhanced therapeutic ambition of triplet immuno-metabolic therapy be translated safely into the clinic.

### 7.3 Sequential and Combination Regimen Design: ICI+TKI+Metabolic; ICI+Radiotherapy+Metabolic; Local Therapies (TACE/HAI/RFA) Combined with Metabolic Modulation

The integration of ICIs with tyrosine kinase inhibitors (TKIs), radiotherapy, and metabolic modulators represents a multifaceted strategy aimed at overcoming the immunosuppressive TME in CCA. For example, ICIs combined with anti-angiogenic TKIs such as lenvatinib or FGFR inhibitors modulate tumor vasculature and immune infiltration, thereby enhancing immune cell access and checkpoint efficacy. Radiotherapy adds further benefit by inducing immunogenic cell death, releasing tumor-associated antigens and up-regulating major histocompatibility complex (MHC) expression, thereby priming the immune response (169). When metabolic inhibitors (e.g., targeting glycolysis, fatty acid oxidation, or amino acid metabolism) are added to these combinations, there is potential for modulating nutrient depletion, reducing immunosuppressive metabolites, and shifting the TME from “cold” to “hot” (170). Concurrently, local therapies such as transarterial chemoembolization (TACE), hepatic arterial infusion (HAI), and radiofrequency ablation (RFA) serve to debulk tumor burden, improve antigen release and reduce the suppressive stroma, setting the stage for systemic immunometabolic therapies to act more effectively. Recent retrospective and prospective data in advanced intrahepatic CCA show that local–regional therapy plus systemic ICI/TKI regimens can achieve objective response rates exceeding 60% and median OS beyond 20 months (171). The addition of metabolic modulators in such regimens is currently under investigation and shows promise in early-phase studies. Collectively, the design of sequential and combination regimens that leverage tumor debulking, vascular and metabolic remodeling, and immune reinvigoration provides a robust framework for next-generation therapeutics in CCA.

### 7.4 Drug delivery and pharmacokinetics: liver and biliary targeted nanodelivery, pH/ROS/Bile Salt Responsive Systems, Local Infusion Strategies

In recent years, liver- and biliary-targeted nanodelivery systems have gained prominence for delivering metabolic inhibitors and immune-checkpoint inhibitors (ICIs) directly to the hepatobiliary compartment, thereby improving tumor specificity and reducing systemic toxicity (172). For example, nanoparticle platforms functionalized with bile-acid transporters or cholangiocyte-specific ligands enable selective uptake by bile-duct epithelial and CCA cells, enhancing local drug concentration in the biliary tree. Stimuli-responsive systems further refine delivery: pH-sensitive linkers release cargo in the acidic TME, ROS-responsive nanoparticles exploit elevated oxidative stress for triggered drug release, and bile-salt-responsive carriers leverage the high bile-salt milieu of the biliary system for enhanced payload liberation. Recent preclinical reports in CCA and liver cancers demonstrate that such smart carriers increase tumor accumulation, extend circulation half-life, and improve internalization into malignant cells (173). Moreover, local infusion strategies—such as hepatic artery infusion (HAI) or intraductal catheter-based delivery—are now being combined with nanocarrier-based metabolic therapies to achieve high local drug exposure, low off-target effects, and better immune modulation (174). For instance, targeted nanovesicles loaded with siRNA against PD-L1 in CCA cells improved T-cell cytotoxicity when delivered via EpCAM-directed carriers, highlighting the translational potential of nanoscale delivery in biliary tumors (175). Together, these advances in pharmacokinetics and delivery systems at the metabolism-immunity interface pave the way for next-generation therapeutic strategies in CCA and BTCs.

### 7.5 Safety and management: hepatobiliary toxicity, metabolic side effects, immune-related adverse events (irAEs) interactions and monitoring

As with any combination therapy in the hepatobiliary context, close attention must be paid to hepatobiliary toxicity, metabolic side-effects, and immune-related adverse events (irAEs) in order to optimise therapeutic outcomes (176). The use of metabolic inhibitors—particularly those targeting glucose or lipid metabolism—carries risks including hepatotoxicity, elevations in liver enzymes, steatosis or steatohepatitis, gastrointestinal disturbances, and metabolic derangements such as hyperglycaemia or dyslipidaemia. Recent evidence also shows that ICI-induced hepatobiliary injury may present not only as classic hepatitis but also as immune-related cholangitis or sclerosing-cholangitis-like picture, especially in biliary cancers and in patients with underlying liver disease (177). For instance, ICI-induced cholangitis, although rare, is increasingly reported and may be severe, with intrahepatic ductal damage and bile-duct dilatation requiring high-dose steroids. From a monitoring perspective, baseline liver assessment, serial hepatic biochemistry (ALT, AST, ALP, GGT, bilirubin), bile-duct imaging when indicated, and prompt referral to hepatology are imperative. In addition, when combining metabolic therapies with ICIs, overlapping toxicities may amplify risk: e.g., mitochondrial inhibitors may exacerbate immune-mediated liver injury via increased oxidative stress. Thus, proactive management includes pre-treatment risk stratification (e.g., underlying cirrhosis, cholestasis), early detection of grade ≥ 2 hepatobiliary events, and algorithm-driven use of immunosuppression or therapy interruption (178). Finally, metabolic side-effects (such as insulin resistance, dyslipidaemia) and immune-related endocrine, dermatologic or gastrointestinal irAEs must be integrated into the safety monitoring plan. Tailoring treatment regimens—dose adjustment, sequential rather than concurrent therapy, and biomarker-driven patient selection—are crucial to balancing efficacy with safety in complex, combined immuno-metabolic strategies in CCA.

## 8 Biomarkers & patient selection

### 8.1 Genomic/epigenomic: MSI-H/TMB, driver mutations, and immune-metabolic signatures

Although microsatellite instability-high (MSI-H) and high tumor mutational burden (TMB-H) are relatively rare in CCA, occurring in approximately ~1–5% of cases, their presence aligns with increased PD-L1 expression and markedly improved responses to ICIs. For example, a 2024 large-cohort study of 887 CCA patients found that MSI-H tumors had a median TMB of ~41.7 mut/Mb (versus ~3.1 mut/Mb in MSS) and a higher PD-L1 positivity (37.5% vs. 11.9%), and those patients experienced significantly longer OS and progression-free survival (PFS) under PD-1 inhibitor-based therapy (179). Beyond MSI/TMB, driver mutations such as IDH1/2, FGFR2 fusions, KRAS, TP53 and BAP1 are far more common in CCA, and carry both prognostic and therapeutic implications. For instance, IDH1/2 mutations create a hypermethylated “non-inflamed” immune microenvironment by generating D-2-hydroxyglutarate and suppressing antigen-presentation machinery, while FGFR2-altered tumors often show lower immune infiltration (180). Importantly, recent multi-omics analyses have begun to integrate genomic, epigenomic, immune- and metabolic-signatures into comprehensive stratification frameworks. A 2024 study distinguished distinct metabolic subtypes of CCA, linking driver gene profiles, metabolic enzyme expression, DNA-methylation patterns and immune-infiltration scores (181). These integrated signatures capture how alterations in metabolic wiring (e.g., sterol/cholesterol handling, amino-acid metabolism) can feed back to epigenetic silencing or activation of immune-related genes (182). Hence, while MSI-H and TMB-H remain minority biomarkers in CCA, driver-mutation status combined with immune-metabolic profiling offers a far richer framework for patient stratification and tailored therapy. Integrating genomic and epigenomic profiling with immune/metabolic signatures enables a more precise, stratified approach to matching patients with ICI, targeted and metabolic-modulation therapies.

### 8.2 Transcriptomic and proteomic: immune inflammation scores and metabolic signatures

Recent integrative transcriptomic and proteomic studies in CCA have elucidated how immune-inflammation scores and metabolic-gene signatures co-associate with tumour microenvironment (TME) states, prognosis, and therapy response. Immune-inflammation scores—derived from gene-expression modules of interferon-γ signalling, cytotoxic T-cell activity, antigen-presentation and immune-checkpoint expression—have been found to segregate “immune-hot” versus “immune-cold” CCA phenotypes, with the former associated with better OS and improved response to ICIs.

In parallel, metabolic signatures involving glycolysis (e.g., LDHA), FAO/lipogenesis (e.g., FASN, SCD1) and mitochondrial dysfunction (e.g., an OXPHOS gene set) correlate strongly with adverse clinical features in intrahepatic CCA (iCCA). For example, a six-gene lactate-metabolism signature was validated in iCCA and showed high lactate-/glycolysis-score tumours had worse survival outcomes (183). Another study classified mitochondrial-dysfunction signatures into two iCCA subtypes—patients with higher mitochondrial signature exhibited significantly lower CD4^+^ T-cell, NK-cell, and monocyte infiltration, and worse prognosis (184). When immune-inflammation scores are combined with metabolic signatures, stratification improves: tumours with high metabolic-signature/high immune-cold scores fare poorest, while low metabolic/high inflammation scores fare best. Further, proteomic profiling in BTCs identified metabolic-subtype tumours enriched for hypoxia/agent resistance pathways and immune-suppressed microenvironments (185). Together, these findings support the view that integrating transcriptomic immune-inflammation scores with metabolic-gene/protein signatures provides a more nuanced patient stratification framework in CCA, enabling better prediction of therapy response and informing personalised treatment strategies.

### 8.3 Metabolomics/lipidomics/bile acid profiling and kynurenine/tryptophan ratio

Metabolomic and lipidomic analyses in CCA have uncovered distinctive metabolic signatures, with particular emphasis on perturbed bile-acid metabolism. For instance, a comprehensive serum metabolome-wide association study in CCA patients identified markedly elevated levels of secondary bile acids (including lithocholic acid [LCA] and taurocholic acid [TCA]) and conjugated bile acids, which were strongly linked to advanced disease stage and poorer survival outcomes (186). Experimental data show that these aberrant bile acids may promote cholangiocyte proliferation, trigger pro-inflammatory signalling, and modify immune cell infiltration, underscoring the immunometabolic impact of the biliary niche. Meanwhile, the kynurenine/tryptophan (Kyn/Trp) ratio has emerged as a robust biomarker of immune suppression: recent studies demonstrate that elevated Kyn/Trp ratios correlate with increased IDO or tryptophan-2,3-dioxygenase (TDO) activity, heightened regulatory T-cell (Treg) infiltration, impaired CD8^+^ T-cell function, and worse clinical outcomes across hepatic malignancies (187). In CCA specifically, spatial transcriptomic work shows that tumour-derived tryptophan metabolism restricts tertiary lymphoid structure (TLS) formation and fosters an immunologically “cold” microenvironment (188). Integrating bile acid/lipid-metabolite profiling with Kyn/Trp ratios thus yields a powerful dual-axis biomarker platform reflecting both metabolic rewiring and immune-evasion. This profiling can stratify patients into high-risk metabolic-immunosuppressive phenotypes (high bile-acid signature + high Kyn/Trp) vs lower-risk phenotypes, thereby guiding selection for combined metabolic–immune therapies. Together, these findings support the routine incorporation of comprehensive metabolomics, lipidomics and tryptophan-catabolism assays into CCA clinical studies to solidify biomarker-driven, personalized treatment strategies.

### 8.4 Radiomics and novel tracers: FAPI-PET and liver-specific contrast agents

Radiomic and advanced molecular imaging techniques are rapidly enhancing the detection and characterization of CCA. Among them, imaging with fibroblast-activation-protein-inhibitor positron emission tomography (FAPI-PET) has emerged as a particularly promising tracer. Studies show that FAPIs target the stromal component (CAFs) of desmoplastic tumours like CCA, offering superior sensitivity and lesion delineation compared to traditional 18F-FDG PET. For example, a 2024 review reports that FAPI-PET frequently outperforms FDG in detecting lesions in cancers with pronounced stromal reaction (189). In CCA specifically, immunohistochemical studies identified high expression of FAP in tumour stroma, strengthening the rationale for FAPI imaging in these patients (190). Early clinical comparative studies of FAPI-PET vs FDG PET demonstrate higher tumour-to-background ratios (TBR), improved detection of small or well-differentiated lesions, and better delineation of both primary lesions and metastases (191). Beyond PET tracers, radiomic analysis of liver imaging, especially MRI using hepatocyte-specific contrast agents (e.g., gadoxetate disodium), is increasingly used to capture subtle features of CCA. These liver-specific contrast agents enhance the hepatobiliary phase imaging, revealing tumour vascular patterns, biliary infiltration, and stromal features more clearly than standard contrast (192). Recent radiomics studies, including a 2025 machine-learning model based on DCE-MRI radiomics, achieved AUC ~0.93 for differentiating iCCA from HCC and other liver lesions, highlighting the power of imaging-feature extraction in CCA (193). Taken together, the integration of FAPI-PET (which captures stromal and microenvironmental biology) with liver-specific contrast enhanced MRI radiomics (which extracts high-dimensional imaging features of tumour structure, vascularity and biliary involvement) offers a new, powerful framework for CCA staging, treatment planning and response monitoring. These advances suggest that radiomics plus novel tracers may soon shift the paradigm for imaging in CCA from purely anatomical to truly functional, microenvironment-aware diagnostics.

### 8.5 Liquid biopsy: cfDNA/ctDNA-MRD, hGE dynamics, exosomes, and circulating metabolites

Liquid biopsy—encompassing analysis of cfDNA, ctDNA, exosomes and circulating metabolites—has rapidly emerged as a minimally invasive strategy for monitoring tumour burden, treatment response and minimal residual disease (MRD) in CCA. Recent reviews and studies show that dynamic changes in ctDNA levels correlate with tumour loads, metastatic potential and therapeutic efficacy; for example, lower post-treatment ctDNA levels in CCA are associated with longer progression-free survival (194). Beyond peripheral blood, bile and duodenal fluid have been demonstrated as valuable liquid-biopsy matrices in CCA: a 2025 study showed that bile samples reliably preserved ctDNA and protein biomarkers for up to 7 hours at room temperature, improving logistics for sample collection in biliary tumours (195). Additionally, exosome-derived nucleic acids and metabolites mirror metabolic rewiring in CCA and may serve as early biomarkers of disease progression or treatment resistance (196). Crucially, high-quality biomarker discovery depends on rigorous sample acquisition and processing protocols: standardisation of collection time points, fluid type (blood vs bile), pre-analytical handling (centrifugation, storage temperature), and multi-site/temporal sampling are essential to minimise variability and support reproducibility in multi-omics analyses (197). Thus, integrating liquid-biopsy readouts with tissue, bile and blood sampling, alongside standardised protocols, offers a comprehensive platform for real-time tracking of the immune-metabolic landscape in CCA.

### 8.6 Spatial biomarkers: immune-metabolic co-localization indicators

Emerging spatial multi-omics technologies—including ST, spatial metabolomics and multiplex imaging—are illuminating the intricate spatial architecture of the tumour microenvironment (TME) in iCCA. For example, a 2024 study applied ST in iCCA and found that TAMs dominate the immune infiltrate, and that tumour cell subclones with high expression of the secreted factor TFF3 co-localize with CD68^+^ macrophages in stromal niches, contributing to an immunosuppressive microenvironment (128). Similarly, a 2025 integrative spatial-metabolomic/transcriptomic investigation in hepatobiliary tumours revealed that malignant cell clusters with a “metabolism archetype” (characterised by up-regulation of lipid-metabolism and oxidative-phosphorylation gene modules) spatially co-localised with TREM2^+^ TAMs and were spatially segregated from CD8^+^ T-effector populations (198). These data support the concept that spatial proximity of metabolic-reprogrammed tumour cells with immunosuppressive myeloid niches fosters immune exclusion. In iCCA specifically, spatial mapping of the “leading-edge” tumour region (bordering non-tumour tissue) by single-cell + ST found that tumour cells enriched in the edge area were tightly associated with stroma (POSTN^+^/FAP^+^ fibroblasts) and endothelial cells, while CD8^+^ T cells in these same zones exhibited an exhausted phenotype with low cytotoxicity, likely reflecting hostile metabolic/immune micro-niches (125). Building on these findings, the spatial co-localisation of immune cell subsets (e.g., macrophages, plasmablasts) with metabolic features (e.g., FAO, lactate export, hypoxia signalling) emerges as a powerful prognostic indicator: tumours with high immune-metabolic co-localisation scores (for example, TAMs + high lipid-metabolism signature) are associated with worse progression-free survival, whereas setups showing effector T cells in low-metabolism zones correlate with better outcomes. Consequently, spatial biomarkers that capture immune-metabolic neighbourhoods—rather than simply immune-cell density or bulk metabolic signature—provide a refined stratification tool. These spatial maps can guide tumour region-of-interest (ROI) in image-guided biopsies, inform therapeutic decisions (for example, targeting metabolic pathways in TAM-rich regions), and serve as dynamic read-outs for therapy response monitoring. As technological platforms mature, translating spatial immune-metabolic co-localisation into clinical stratification and biomarker‐driven trials in CCA holds significant promise.

### 8.7 Predictive and monitoring frameworks: baseline stratification, early dynamic response, and timely sequencing

A robust biomarker-based framework for managing CCA requires three interlinked components (1): baseline stratification (2), early dynamic response monitoring, and (3) timely sequencing of therapies. At baseline, multiplex profiling — including genomic (e.g., driver mutations, MSI/TMB), transcriptomic (immune-inflammation scores), metabolic (metabolite/lipid signatures) and imaging/radiomics data — enables identification of patient subgroups most likely to benefit from specific therapeutic modalities. Recent reviews emphasise the importance of such multi-omics baseline stratification in CCA to avoid “one-size-fits-all” approaches and to direct combined metabolic-immunotherapy more precisely (123). Following treatment initiation, early dynamic monitoring via serial liquid biopsies (ctDNA changes, circulating metabolites, exosomes) and imaging (functional PET/MRI) allows assessment of therapeutic efficacy within weeks rather than months. For example, liquid‐biopsy studies across GI cancers highlight how early declines in ctDNA correlate with response and permit early switching of non-responders (199). In CCA specifically, emerging data suggest that real-time biomarker shifts (e.g., drop in ctDNA, metabolite signature reversion) precede radiographic response and can trigger earlier therapy adjustment, although more prospective evidence is needed. Finally, timely sequencing of treatment regimens — guided by this real-time data — enables adaptive adjustments: escalating combination regimens (e.g., adding metabolic inhibitors) for early non-responders, or de-escalating toxicity for early good responders. Analytical frameworks now propose integrating real-time biomarker kinetics and imaging changes into decision algorithms (200). Together, this triple-axis framework transforms static stratification into a dynamic, adaptive therapeutic model — ideal for precision medicine in CCA — enabling more effective personalization of therapy, earlier detection of resistance, and improved outcome optimization.

### 8.8 Novel genomic-metabolic biomarkers

In CCA immunotherapy, the interaction between metabolic reprogramming and the immune microenvironment is increasingly recognized as a critical factor influencing tumor progression and treatment response. Recent studies have found that high expression of PGM2L1 in CCA is associated with poor prognosis. Wu et al. (2025) reported that as a key enzyme in glycolysis/glycogen metabolism (catalyzing G1P/G6P ↔ G1,6BP), PGM2L1 may affect CCA progression by promoting metabolic reprogramming and modulating the immune microenvironment (201). Additionally, the high expression of GPD2 (mitochondrial glycerol-3-phosphate dehydrogenase 2) in CCA is closely related to tumor cell proliferation and migration. Its mechanism of action includes regulating glycolysis and lactate accumulation, thereby influencing immune cell function in the TME. Knockdown of GPD2 can inhibit CCA cell proliferation and enhance CD8^+^ T-cell anti-tumor immune responses (202). Furthermore, the ANXA1-USP5-GOT1 axis plays an important role in metabolic reprogramming. Upregulation of ANXA1 stabilizes USP5, which further enhances glutamine metabolism and alters the TIME, particularly by promoting the polarization of M2 macrophages and inhibiting T-cell anti-tumor activity (203). In addition, metabolomics studies have revealed that the levels of metabolites such as LysoPCs (lysophosphatidylcholine) and PCs (phosphatidylcholine) are significantly altered in the serum of CCA patients. These metabolites are closely associated with tumor glycolysis and lipid metabolism and may serve as new liquid biopsy biomarkers (204).

### 8.9 Rational biomarker development

Despite advances in the field of metabolic-immune therapeutic strategies for CCA, the development of clinically validated biomarkers remains a major hurdle. Emerging studies highlight that liquid-biopsy tools such as circulating tumour DNA (ctDNA) and cell-free DNA (cfDNA) hold promise for non-invasive monitoring, yet they still lack large-scale prospective validation and remain disconnected from spatial heterogeneity of tumour metabolism or immune microenvironment (194). Furthermore, metabolomics and lipidomics analyses have identified distinct signatures (e.g., carnitines, glycerophospholipids) associated with CCA recurrence and patient outcomes, but these too confront challenges of reproducibility and standardisation across platforms (205). Finally, for practical translation into stratified therapy, ideal biomarkers must be minimally invasive, repeat-monitorable, and directly actionable in treatment decisions; however, most candidates remain in an exploratory stage and are not integrated into clinical workflows for metabolic-immune combination therapy (197).

## 9 Clinical evidence & trials

### 9.1 Key milestones in ICI monotherapy and chemotherapy/targeted therapy combinations in BTC

ICIs have shown promise in BTC, particularly in combination with chemotherapy (206). The TOPAZ-1 trial demonstrated that the addition of durvalumab to gemcitabine and cisplatin (GemCis) improved OS in advanced BTC patients. Similarly, the KEYNOTE-966 trial with pembrolizumab plus GemCis also reported enhanced survival outcomes. Real-world studies have corroborated these findings, showing improved PFS and OS with ICI plus chemotherapy regimens compared to chemotherapy alone. However, the incidence of grade 3–4 AEs remains a concern, necessitating careful patient selection and monitoring (207).

### 9.2 Human exploration of metabolic modulation strategies: published and ongoing trials

Metabolic reprogramming is a hallmark of CCA, influencing tumor growth and immune evasion. Targeting metabolic pathways, such as fatty acid metabolism, has emerged as a therapeutic strategy. Recent studies have highlighted the role of fatty acid metabolism in CCA progression, suggesting that inhibiting enzymes involved in this pathway could suppress tumor growth. Ongoing clinical trials are investigating the efficacy of combining metabolic modulators with ICIs to enhance anti-tumor immunity. For instance, a study combining chemotherapy, ICIs, and electro-hyperthermia reported positive outcomes in treating local lymphatic recurrence of CCA (208) (**Table 1**).

**Table 1:** Clinical trials of drugs metabolized by cholangiocarcinoma.

Trial ID	Phase (Estimated number); line	Tumor type	Molecular target	Treatment type	Status	Last update posted	Primary outcome	Location
NCT04203160	I(N = 75); 1st	GBC	N	CPI 613 Cisplatin Gemcitabine	Completed	7/11, 2024	ORR	US
NCT02989857	III(N = 187);1st	CCA	IDH1	AG-120 Placebo	Completed	8/20, 2024	mPFS	US
NCT04163900	III(N = 773);1st	BTC	N	NUC-1031 Gemcitabine Cisplatin	Completed	5/25, 2023	mOS	US
NCT04088188	I(N = 8)1st	CCA	IDH	Ivosidenib cisplatin gemcitabine	Completed	5/6, 2024	Maximum tolerated dose	US
NCT02496741	Ib(N = 15)1st	iCCA	IDH1/2MT	Metformin and chloroquine combination	Completed	1/9, 2020	Maximum tolerated dose	US
NCT02381886	I(N = 166)1st	BTC	IDH1R132	IDH305	Completed	8/29, 2025	Dose limiting toxicities	US
NCT01525719	II(N = 40)1st	BTC	mTOR	Everolimus	Completed	2/3, 2012	PFS	Thailand
NCT03377179	IIA(N = 65)1st	CCA	N	ABC294640 Hydroxychloroquine Sulfate 200 MG	Completed	7/23, 2016	ORR	US
NCT04521686	I(N = 200)2nd	CCA	R172	LY3410738 Gemcitabine Cisplatin Durvalumab	Active, not recruiting	6/27, 2025	Recommended phase II dose	US
NCT03684811	Ib(N = 93)3rd	BTC	IDH1	FT-2102 Azacitidine Nivolumab Gemcitabine Cisplatin	Completed	11/18, 2023	Dose limiting toxicity, recommended phase II dose	US
NCT01766219	I-II(N = 17)2nd	CCA	CPI-613	1,200-3,000 mg/m^2^ dose-escalation, 28-day cycle	Completed	5/15, 2019	OS	US
NCT00949949	II(N = 217)1st	CCA	N	Tucatinib; Trastuzumab; Fulvestrant	Active, not recruiting	1/12, 2017	Adverse events profile, toxicity profile, MTD	US

iCCA, Intrahepatic Cholangiocarcinoma; BTC, Biliary Tract Cancer; ORR, Overall Response Rate; PFS, Progression-Free Survival; OS, Overall Survival; N: None.

Recent clinical trials in advanced CCA and BTC have reported incremental improvements with ICIs or targeted therapies—for instance, the phase 3 TOPAZ-1 trial showed a statistically significant OS benefit when adding Durvalumab to gemcitabine/cisplatin (177). Nonetheless, significant evidence gaps and limitations constrain broader clinical translation. First, many trials enrol highly selected patient populations (e.g., with specific molecular alterations or good performance status) that do not reflect real-world heterogeneity, and sample sizes remain modest (209). Second, the complexity of the tumour immune−metabolic microenvironment in CCA—characterised by desmoplasia, metabolic reprogramming, immune exclusion and spatial heterogeneity—limits the predictive value of early results and complicates extrapolation of preclinical models (210). Third, many combination strategies (e.g., ICI + metabolic-targeting agents) lack mature long-term outcome data, particularly in terms of durability of response, second-line efficacy and biomarker-driven patient selection. Finally, many trials inadequately report key methodological details—such as biomarker stratification, sample size justification, selection criteria and statistical endpoints—reducing comparability and reproducibility across studies (211). Collectively, these limitations underscore the urgent need for larger, biomarker-guided trials with rigorous design, comprehensive correlative studies and longer follow-up to translate mechanistic promise into meaningful clinical impact in CCA.

### 9.3 Safety, side effects, and monitoring strategies for combination therapies

Combination therapies in advanced CCA — particularly regimens integrating chemotherapy, ICIs and metabolism-targeting agents — have shown encouraging efficacy, but their safety profile demands rigorous attention. Recent studies report that while high-grade AEs appear manageable, the cumulative burden of hepatobiliary and immune-related toxicities is non-trivial given the liver-centric milieu of CCA. Real-world pharmacovigilance data indicate a predominance of endocrine and hepatobiliary disorders emerging early after ICI combinations (212). Mechanistic reviews highlight that hepatic injury from ICIs may be mistaken for disease progression or cholestasis, underscoring the need for differentiated monitoring (213). In recent multicentre cohorts combining local-regional therapy plus chemotherapy plus ICI plus targeted agents in intra-hepatic CCA, grade 3–4 AE incidence reached ~66 %, including myelosuppression, AST/ALT elevations and fatigue, but no grade 5 events and overall toxicities were deemed manageable (169). From a monitoring strategy perspective, this calls for baseline assessment of liver function (ALT/AST, ALP, bilirubin), dynamic metabolic panels, immune-related parameters (e.g., T-cell subsets, cytokines) and bile-duct injury biomarkers; early-onset AEs within the first 4–8 weeks should trigger intensified surveillance and prompt intervention (e.g., corticosteroids for immune toxicity, dose-modification for metabolic agents). Notably, future trials must prospectively stratify patients by hepatic reserve, underlying cholestasis or biliary obstruction, and define predefined AE stopping rules for metabolism/immune-therapy overlap. Without such safety architecture, the translational promise of immuno-metabolic “triplet” or “quadruplet” regimens in CCA risks being compromised.

### 9.4 Evidence gaps: real-world data, translational cohorts, and companion diagnostics

Real-world data (RWD) are limited, particularly concerning the long-term efficacy and safety of combination therapies. Translational cohorts are essential for understanding the molecular underpinnings of treatment responses and resistance mechanisms. Furthermore, the lack of standardized companion diagnostics hampers the ability to tailor therapies to individual patients effectively. Addressing these gaps through comprehensive RWD studies, biomarker discovery, and the development of companion diagnostics is crucial for advancing personalized treatment approaches in CCA (214).

## 10 Methodological considerations

### 10.1 Translational research pathways: from tissue to single-cell, spatial, metabolic, and clinical validation

Modern research in iCCA increasingly adopts an end-to-end translational workflow that spans tissue analysis, single-cell profiling, spatial omics, metabolic evaluation and clinical validation. At the tissue level, bulk and proteogenomic studies have identified major driver alterations and stromal signatures, but the real breakthrough has been through single-cell RNA-sequencing (scRNA-seq). For example, a 2025 study found that tumour cells expressing high levels of SPP1 co-operate with CD4^+^ T-cells through a SPP1-CD44 axis and are associated with poor prognosis in iCCA (215). Beyond transcriptomics, integrative single-cell multi-omics reviews show that combining genomics, epigenomics and transcriptomics uncovers tumour-stromal heterogeneity and immune microenvironment remodeling in BTCs (216). Spatial omics then adds the dimension of where in the tissue particular cell states and metabolic programs exist. A 2024 ST study revealed niche-specific immune-stromal interactions in CCA, indicating that spatial neighbourhoods of metabolic-reprogrammed tumour cells and immunosuppressive macrophages form functional units driving progression (128). On the metabolic side, metabolomic/lipidomic profiling of tumour tissue or adjacent bile has permitted identification of metabolic signatures (e.g., glycolysis, FAO) that align with immune phenotypes and clinical outcomes. For example, a 2024 multi-omics study of CA19-9-positive iCCA linked glycolysis-associated cell clusters with poor survival and provided a basis for precision therapeutic stratification (217). Critical to translation is clinical validation: tissue and liquid biopsy biomarkers must be correlated with patient outcomes, and then used to guide prospective trials. The integration of multi-omics (single-cell + spatial + metabolic) with clinical endpoints enables the identification of therapeutic biomarkers and predictive signatures that can guide enrolment of patients into targeted metabolic–immune therapy combinations. In sum, this translational cascade — from tissue to single-cell to spatial to metabolic to clinic — provides the framework by which mechanistic discoveries are moved into validated biomarkers and actionable treatment strategies for iCCA.

### 10.2 Statistical endpoints and adaptive trial designs: composite endpoints (PFS/OS + Metabolic-Immunological Biomarkers), Adaptive Trial Designs

In designing clinical trials for CCA, relying solely on traditional endpoints such as PFS and OS may not fully capture the multifaceted effects of novel therapies—especially those targeting the immuno-metabolic axis. Therefore, incorporating composite endpoints that blend PFS/OS with validated metabolic and immunological biomarkers has become increasingly important. For example, a 2024 biomarker study in BTCs developed an immune/metabolism score based on six genes, which correlated with response to immunotherapy, suggesting that biomarker-enhanced endpoints can identify benefit beyond survival curves (218). Such composite endpoints may include metrics like change in ctDNA, shifts in metabolite profiles (e.g., Kyn/Trp ratio), or immune cell infiltrate changes, combined with time-to-event data, thereby improving sensitivity and enabling shorter trial durations.

In parallel, adaptive trial designs are gaining traction in oncology and are particularly suited for CCA where patient populations are small and heterogeneity is high. These designs allow interim modifications based on accumulating data—such as enrichment of biomarker-positive subgroups, early stopping for futility/efficacy, dynamic dose adjustment, or arm dropping. For instance, biomarker-guided adaptive enrichment designs have been proposed to improve estimation of treatment effects in subpopulations defined by metabolic–immune status (e.g., high lipid-metabolism + immune-cold phenotype) (219). In CCA, applying such designs means that patients can be stratified early by baseline biomarker profiles, and the trial can adapt by narrowing to those most likely to respond or switching regimens if early biomarker dynamics (e.g., ctDNA drop, metabolite signature change) indicate non-response.

Together, the combination of composite endpoints that integrate metabolic/immunological biomarkers with survival outcomes, plus adaptive trial architectures, offers a more efficient and informative strategy for CCA trials. This approach allows earlier decision-making, better resource allocation, and more rapid identification of effective therapeutic combinations in this challenging tumor setting.

### 10.3 Data integration and AI: multi-omics, imaging, and clinical data for interpretable modeling

Integrating multi-omics data with imaging and clinical information is pivotal for developing predictive models in CCA. Artificial intelligence (AI) techniques, such as machine learning and deep learning, facilitate the analysis of complex datasets, identifying patterns and correlations that may not be apparent through traditional methods. For instance, a study integrating radiomics and multi-omics data developed a predictive nomogram for improved prognosis in CCA patients (220). Additionally, AI applications in CCA have demonstrated potential in early detection, prognosis prediction, and treatment response assessment (221). Ensuring the interpretability of AI models is crucial for their clinical adoption, requiring transparent algorithms and validation across diverse patient cohorts.

## 11 Controversies & open questions

### 11.1 IDO inhibitors: pathway selection post first-generation failures

ICI therapy and emerging metabolic-targeting approaches have recently begun to alter the therapeutic landscape for advanced CCA, with landmark trials such as the first-line addition of Durvalumab to gemcitabine/cisplatin demonstrating statistically significant improvement in OS in BTC/CCA patients (222). However, despite these promising advances, numerous trials have failed to achieve their endpoints or have shown only modest benefit—highlighting key translational hurdles. For instance, early trials of first-generation IDO inhibitors in combination with ICIs did not meet pre-specified overall response rate (ORR) thresholds and were terminated due to insufficient efficacy. Moreover, many ICI trials in CCA/CBTC enrolled small heterogeneous cohorts, lacked biomarker-based stratification, and demonstrated only modest PFS and OS benefits (e.g., ORR ~13 %, median PFS ~2.5 months in some cohorts) (178). These outcomes underscore the need for metabolic and immune biomarker-driven patient selection, improved trial design, and deeper interrogation of resistance mechanisms (e.g., tumour metabolic heterogeneity, immune exclusion, stroma-mediated sequestration) to translate mechanistic promise into clinical benefit in CCA.

### 11.2 Safety window and immune consequences of FAO inhibition

Fatty acid oxidation (FAO) is increasingly recognised as a critical energy-producing pathway not only for tumour cells but also for immunosuppressive myeloid and regulatory immune cells in the TME. For example, in CCA, a recent review reports that FAO is the preferred energy source for proliferating CCA cells compared to normal bile duct epithelium, and pharmacologic blockade of FAO suppresses tumourigenicity in pre-clinical CCA models (113). In immune cell populations, FAO supports the function and survival of tumour-associated macrophages (TAMs), MDSCs and Tregs. Inhibiting FAO has been shown to impair the immunosuppressive functions of these cells — for instance, FAO inhibition in MDSCs reduced suppressive cytokine release, decreased Treg induction, and enhanced CD8^+^ T cell-mediated anti-tumour responses in model systems (223). However, the safety window for FAO inhibition remains a significant concern. Because FAO is also important for memory CD8^+^ T cell formation and normal tissue homeostasis, excessive suppression may lead to unintended consequences such as immune dysregulation, impaired T cell memory formation, metabolic toxicity (e.g., hepatosteatosis, myocyte dysfunction) or exacerbation of metabolic comorbidities (224). Therefore, balancing therapeutic efficacy with safety is critical. Ongoing studies emphasise the importance of defining optimal dosing regimens, duration of inhibition, and patient stratification via biomarkers predictive of benefit and risk. For example, selecting patients whose tumour and immune microenvironment show high FAO dependence (via elevated CPT1A, ACAD, or CD36 expression) may maximise benefit while monitoring liver enzymes, lipid profiles, and T cell memory markers may enhance safety (225). In sum, while FAO inhibition offers a promising strategy to both block tumour energy supply and relieve immunosuppression, its implementation requires careful calibration of dose, timing, patient selection and biomarker-guided monitoring to ensure the “therapeutic window” is both effective and safe.

### 11.3 Bidirectional effects of the bile acid axis: dose, timing, and population heterogeneity

Bile acids (BAs) and their receptors represent a highly complex and context-dependent axis in CCA pathogenesis and therapy. On one hand, at physiological or moderately elevated concentrations, certain BAs activate receptors such as Farnesoid X Receptor (FXR) which may exert tumour-suppressive effects by restraining cholangiocyte proliferation and maintaining biliary homeostasis. Early work demonstrated that FXR activation inhibited CCA cell growth, while the G-protein-coupled receptor TGR5 (GPBAR1) was upregulated in CCA and associated with more aggressive behaviour (24). On the other hand, in conditions of high BA load, cholestasis, or dysregulated enterohepatic cycling, BAs accumulate and can engage alternative receptor and non-receptor mechanisms to promote tumour progression. For instance, secondary and conjugated BAs may activate inflammatory and fibrogenic signalling (e.g., via NF-κB, IL-6/STAT3), induce oxidative stress, and create an immunosuppressive microenvironment through modulation of the gut-liver axis and bile duct fibroinflammatory responses (154). Importantly, recent studies emphasise the role of dose and timin**g** in shaping these effects. A 2025 mechanistic study found that elevated concentrations of hydrophobic BAs chronically activated TGR5 on CAFs, inducing CXCL10 expression and thereby recruiting immunosuppressive macrophages in the biliary stroma—contrasting effects seen at lower levels (33). Moreover, patient heterogeneity is critical: factors such as underlying liver disease (e.g., cholangitis, PSC), microbiome composition, bile duct anatomy (intrahepatic versus extrahepatic CCA) and BA transporter polymorphisms modulate the BA signalling outcome. For example, dysbiosis of bile and gut microbiota in CCA patients alters BA pools, leading to accumulation of specific BAs that correlate with more advanced stage and worse survival (226). Collectively, these findings underscore that therapeutic modulation of the BA axis is far from a simple “on/off” intervention: the same pathway may yield anti-tumour or pro-tumour effects depending on the concentration, receptor context, tissue microenvironment, and patient background. Therefore, future therapeutic strategies must incorporate careful consideration of BA species, receptor expression (FXR vs TGR5), timing of intervention (e.g., early in cholangiopathy vs advanced disease), and patient stratification (e.g., BA-metabolism phenotype, microbiome status). This nuanced understanding will be essential to harness beneficial effects of BA-based therapies while mitigating risks of fostering tumour progression or immune suppression.

### 11.4 Reversibility and timing of metabolic “lock” in immune exhaustion

In the tumour microenvironment (TME) of CCA, profound metabolic reprogramming in both malignant and immune-cell compartments can lead to a state of immune exhaustion characterised by persistently impaired CD8^+^ T-cell function, reduced cytokine production, and limited memory formation. Recent studies in immunometabolism show that exhausted T-cells (T_ex_) may become locked into a dysfunctional state through metabolic constraints, such as reduced mitochondrial OXPHOS, glucose and amino-acid scarcity, lipid overload, and the accumulation of immunosuppressive metabolites (e.g., adenosine, lactate) (152). Evidence indicates that the timing of interventions is crucial: in early exhaustion phases (progenitor-like T_ex_, TCF1^+^/PD-1^+^), metabolic reprogramming remains at least partially reversible with strategies that restore nutrient uptake, mitochondrial fitness or block immunometabolic checkpoints, thereby improving function and responsiveness to immunotherapy. However, once T_ex_ progress to a terminal epigenetically “locked” state (PD-1^1hr^/TOX^hr^) with closed chromatin, stable metabolic and epigenetic changes strongly limit reversibility (227). In the context of CCA, where nutrient competition is intense and hypoxia/lactate accumulation common, this “metabolic lock” may establish earlier and more firmly than in other tumour types, making the window of opportunity for metabolic re-invigoration narrow. Therefore, therapeutic approaches targeting metabolic pathways (e.g., restoring mitochondrial OXPHOS, inhibiting FAO in suppressive myeloid cells, modulating glycolysis) must be timed to coincide with early T-cell dysfunction rather than once exhaustion is entrenched. Additionally, patient stratification and biomarker monitoring (such as mitochondrial membrane potential in T cells, metabolite signatures, ctDNA dynamics) can help define when to intervene, increasing the chance of immune-recovery while minimising ineffective late interventions. Collectively, these insights emphasise that successful reversal of immune exhaustion in CCA requires not only the right target (metabolic or immunometabolic) but also the right timing and patient context to avoid wasted effort against an irreversible lock-in.

### 11.5 Net effect of metabolic reprogramming induced by local therapies on systemic immunity

Local therapies, such as Transarterial Chemoembolization (TACE), Hepatic Arterial Infusion (HAI), and Radiofrequency Ablation (RFA), not only directly destroy tumor tissue but also induce metabolic reprogramming within the TME, which can potentially impact systemic immunity. These therapies lead to large-scale tumor cell necrosis, the release of tumor-associated antigens (TAA) and damage-associated molecular patterns (DAMPs), activating local immune responses and potentially inducing an “abscopal effect” — immune-mediated regression of distant tumors or micrometastases after local treatment (228). From a metabolic perspective, local treatments alter the tumor region’s oxygenation, nutrient availability, and lactate/metabolic waste accumulation, impacting both tumor and infiltrating immune cells. For instance, RFA, following thermal necrosis, releases endothelial and tumor metabolites, causing a hypoxia/reperfusion response, which induces ROS accumulation, mitochondrial stress, and metabolic pathway rewiring (229). These metabolic changes can promote antigen-presenting cell (APC) activation and CD8^+^ T cell recruitment, enhancing the overall immune response. On the other hand, if excessive necrosis, prolonged hypoxia, or nutrient deprivation occurs, it may instead induce an immunosuppressive environment. For example, the release of high levels of lactate, amine metabolites, or bile acids from necrotic tissue can activate MDSCs, Tregs, or polarize macrophages into the M2 phenotype, inhibiting systemic immune functions (230). Thus, the “net effect” exists in a delicate balance between enhancing overall antitumor immunity and potentially triggering immune suppression. Key variables include: treatment timing (high vs. low tumor burden), treatment intensity and necrosis volume (small vs. large necrosis areas), and the tumor and host metabolic state (e.g., pre-existing liver disease, nutritional status, microbiome disturbances). Literature suggests that to optimize systemic immune benefits while minimizing negative metabolic reprogramming, local therapies should be combined with immune or metabolic therapies (e.g., adding ICIs or metabolic modulators to low lactate load zones after TACE), while monitoring systemic immune markers (such as circulating lactate, ctDNA, immune cell subset dynamics) to adjust subsequent treatments (231). In conclusion, local therapies for CCA not only serve as a means of local control but also present an opportunity to reshape the tumor-immune-metabolism axis. However, their net effects on systemic immunity are yet to be fully quantified, and future studies should concurrently measure local metabolic changes, immune cell dynamics, and distant tumor responses to better define which treatment combinations, when applied, are most beneficial for specific patient populations.

## 12 Future directions & actionable agenda

### 12.1 “Metabolic conditioning” and “identity locking”: stabilizing effector T cell function in specific metabolic niches

Effector T cells (Teffs) adopt distinct metabolic programs that influence their activation, effector function, persistence, and eventual exhaustion within the tumour microenvironment (TME). The recent review by Ma et al. emphasises that cellular metabolism is not only a fuel source but a regulator of T-cell differentiation and fate, integrating nutrient availability, signalling and epigenetics (232). Teff cells ramp up glycolysis, amino-acid metabolism and lipid synthesis to support rapid expansion and effector cytokine production, while memory and long-lived T cells rely more on FAO and mitochondrial OXPHOS. “Metabolic conditioning” refers to the strategy of pre- or co-treating T cells (or modulating their niche) to shape these metabolic programs such that Teffs remain functionally active and avoid early exhaustion. For instance, a recent study showed that T-cell-specific deficiency of the ADP/ATP translocase 2 (ANT2) in mice – thereby forcing mitochondrial metabolic reprogramming – resulted in enhanced activation, increased mitochondrial biogenesis and improved tumour control (233). This highlights how metabolic rewiring can boost effector function and anti-tumour immunity. “Identity locking” refers to stabilising a desirable functional state of effector T cells through metabolic imprinting: once established, certain metabolic-epigenetic states make the T cell less prone to exhaustion or dysfunction. Reviews on T-cell immunometabolism point out that Teffs face nutrient competition, hypoxia and metabolite accumulation (e.g., lactate, adenosine) in the TME, which can drive them toward dysfunctional or exhausted states via altered metabolism and epigenetics (234). Thus, manipulating metabolic pathways (e.g., enhancing OXPHOS, limiting lipid overload, ensuring amino-acid supply) and shaping the niche (e.g., reducing nutrient competition or immunometabolic suppression) may “lock” T cells into a high-fitness effector identity. In the context of CCA, where the TME is particularly hostile—characterised by nutrient scarcity, hypoxia, lactate accumulation and metabolic competition—such approaches may be especially critical. Strategically conditioning T cells to thrive in those niches (for example by enforcing mitochondrial resilience or altering nutrient preferences) could stabilise their identity and extend their anti-tumour functionality. Ultimately, this approach offers a translational route: combining metabolic interventions (e.g., modulating T-cell metabolism before infusion or administering metabolic adjuvants) with immunotherapy may improve T-cell persistence and response. In summary, the convergence of “metabolic conditioning” and “identity locking” offers a promising conceptual and practical framework for stabilising effector T-cell function in specific metabolic niches such as those found in CCA.

### 12.2 “Metabolic bait/trap” strategies: selective targeting of lactate, bile acids, and lipid metabolic vulnerabilities

Tumor cells in CCA frequently exploit a number of metabolic pathways to support growth, survival, immune evasion and therapy resistance. One prominent axis is lactate metabolism: elevated glycolysis leads to lactate over-production and export via monocarboxylate transporters (MCTs). High lactate levels promote immunosuppression through acidosis, histone lactylation, TAM (tumor-associated macrophage) M2 polarization and CD8^+^ T-cell inhibition. Recent reviews highlight that inhibitors of LDHA or MCT1/MCT4 not only reduce tumor-cell growth but also “trap” the immunosuppressive tumour microenvironment and enhance immunotherapy efficacy (39). Secondly, bile acid (BA) metabolism and signalling represent another targetable vulnerability: dysregulated BA pools and receptor signalling (e.g., FXR, TGR5) alter immune and stromal cell behaviour in the gut-liver/biliary axis of CCA, and modulation of BA receptors or transporters provides a metabolic “trap” to disrupt tumour–immune crosstalk (154). Thirdly, lipid metabolic programmes, including de novo lipogenesis, FAO, and membrane phospholipid remodelling, are up-regulated in CCA and correlate with poor prognosis. Lipid-targeting (e.g., FASN, ACC, SCD1 inhibitors) acts as a metabolic trap: impairing tumor lipid supply, altering membrane composition and increasing vulnerability to immune attack. For example, a 2024 lipidomics study in CCA identified glycerophospholipid and fatty-acid signatures enriched in tumour tissue, underscoring therapeutic opportunities (235). Implementing this “bait/trap” concept means using inhibitors or modulators of these metabolic nodes not only to starve tumour cells, but also to convert the TME from immunosuppressive to immunoreactive—thus sensitizing CCA to ICIs or other immunotherapies. These strategies require biomarker-driven patient selection (e.g., high LDHA/MCT expression, BA-metabolism phenotype, lipid-metabolism signature) and combinatorial design (metabolic inhibitor + ICI) to maximize effect. In summary, selective targeting of lactate export/production, bile-acid signalling and lipid metabolic machinery offers a refined therapeutic paradigm in CCA—one that exploits tumour metabolic vulnerabilities, engages immune dormancy reversal, and positions CCA for precision immunometabolic therapy.

### 12.3 Exploration of metabolic-immune pathways mediated by micropeptides and non-coding transcripts

Long non-coding RNAs (lncRNAs) were formerly considered non-coding, but increasingly it is clear that many of them encode small open reading frame (sORF)-derived micropeptides that exert biological functions (236). These micropeptides can influence metabolic and immune pathways in cancer, such as modulating glycolysis, lipid synthesis, mitochondrial function and immune cell signalling. For example, one lncRNA-encoded micropeptide was shown to regulate ATP synthase subunits and thus mitochondrial ATP production, with downstream effects on metabolic stress and tumour cell survival. In another instance, a micropeptide encoded by an lncRNA maintained acetylation of ACLY (ATP-citrate lyase), promoting lipid accumulation and tumour progression (237). Beyond tumour-intrinsic metabolism, micropeptides also affect immune cell behaviour: reviews highlight how certain micropeptides modulate innate immunity, influence inflammasome activation and alter T-cell and macrophage function (238). In the context of CCA, while direct evidence remains limited, lncRNAs have been implicated in CCA progression and immune evasion (239). it is thus plausible that their encoded micropeptides contribute to the immuno-metabolic reprogramming in the biliary niche. For instance, dysregulation of a CCA-associated lncRNA may lead to altered lipid metabolism in tumour cells and suppressive macrophage phenotypes via a micropeptide-mediated mechanism. From a translational perspective, these micropeptides represent novel therapeutic targets: by inhibiting or mimicking specific micropeptides, one can disrupt tumour metabolic support, re-activate immune cell function, or both. Therefore, this section proposes that investigation into lncRNA-encoded micropeptides and other non-coding transcripts offers a promising frontier for decoding and targeting metabolic-immune crosstalk in CCA.

### 12.4 Integration of spatial omics and liquid biopsy for MRD monitoring and adaptive treatment strategies

The integration of spatial omics and liquid biopsy technologies provides a powerful dual-modality platform for monitoring minimal residual disease (MRD) and guiding adaptive therapeutic strategies in CCA. Spatial omics, including ST, proteomics and metabolomics, allow for *in situ* mapping of tumour cells, immune infiltrates, stromal elements and metabolic niches within tissue architecture, thus revealing how residual tumour-immune-metabolic microdomains persist after therapy and may seed relapse. For example, recent reviews highlight how spatial omics can resolve intratumour heterogeneity, therapy-induced niche remodelling and spatially restricted immune-resistant clones (240). Parallel to this, liquid biopsy approaches—particularly circulating tumour DNA (ctDNA), cell-free DNA (cfDNA), exosomes and circulating metabolites—offer non-invasive, serial read-outs of residual disease burden, molecular evolution and treatment response. In BTCs, ctDNA from plasma, bile fluid or duodenal fluid has shown promising diagnostic and prognostic capability and may reflect MRD status after curative or locoregional therapy (241). By combining spatial omics with liquid biopsy, one can link tissue-level residual micro-niches with systemic biomarkers: for instance, spatially mapped immunosuppressive or metabolic “safe-havens” may correspond with persistent ctDNA or circulating metabolite signatures in the blood or bile, thereby enabling early detection of disease persistence or recurrence. This alignment enables adaptive treatment strategies: if liquid biopsy indicates rising ctDNA and spatial mapping suggests residual tumour niche in a particular region (e.g., periductal stroma), the clinician may escalate therapy (e.g., add metabolic inhibitor, ICI or region-targeted therapy) even before radiographic relapse. Reviews in precision oncology emphasise that such multi-modal tracking transforms stratification from static to dynamic, permitting therapy modulation in real time rather than waiting for overt progression (242). In CCA, implementing this combined approach hinges on standardized workflows—such as obtaining tumour and adjacent tissue for spatial omics prior to treatment, serial liquid biopsy at defined timepoints (e.g., post-surgery, post-locoregional therapy), and integration of biomarker trajectories with imaging and clinical data. Current limitations include the lower ctDNA shedding in some CCA patients, need for high-resolution spatial maps in biliary tissues, and the requirement for validated cut-offs for biomarker changes that trigger treatment modification (243). In summary, the convergence of spatial omics and liquid biopsy forms a next-generation framework for MRD monitoring and adaptive management in CCA—enabling early detection of residual disease, timely therapeutic adjustment and potentially improved outcomes.

### 12.5 Synthetic biology approaches to metabolic reprogramming of CAR-T and NK cells

Tumour cells in CCA frequently exploit a number of metabolic pathways to support growth, survival, immune evasion and therapy resistance. One prominent axis is lactate metabolism: elevated glycolysis leads to lactate over-production and export via MCTs. High lactate levels promote immunosuppression through acidosis, histone lactylation, TAM (tumour-associated macrophage) M2 polarisation, and CD8^+^ T-cell inhibition. Recent reviews highlight that inhibitors of LDHA or MCT1/MCT4 not only reduce tumour-cell growth but also “trap” the immunosuppressive tumour-microenvironment (TME) and enhance immunotherapy efficacy (244). Secondly, bile−acid (BA) metabolism and signalling represent another targetable vulnerability: dysregulated BA pools and receptor signalling (e.g., FXR, TGR5) alter immune and stromal−cell behaviour in the gut–liver/biliary axis of CCA, and modulation of BA receptors or transporters provides a metabolic “trap” to disrupt tumour–immune crosstalk (245). Thirdly, lipid metabolic programmes—including de novo lipogenesis, FAO, and membrane phospholipid remodelling—are upregulated in CCA and correlate with poor prognosis. Lipid-targeting (e.g., FASN, Acetyl-CoA carboxylase (ACC), Stearoyl-CoA desaturase 1 (SCD1) inhibitors) acts as a metabolic trap: impairing tumour-lipid supply, altering membrane composition and increasing vulnerability to immune attack. For example, a 2024 lipidomics study in CCA identified glycerophospholipid and fatty-acid signatures enriched in tumour tissue, underscoring therapeutic opportunities. In this strategy, metabolic inhibitors don’t merely starve tumour cells but also alter the TME to favour immune activation. Implementing this “bait/trap” concept means using inhibitors or modulators of these metabolic nodes not only to starve tumour cells but also to convert the TME from immunosuppressive to immunoreactive—thus sensitizing CCA to immune-checkpoint inhibitors (ICIs) or other immunotherapies. These strategies require biomarker-driven patient selection (e.g., high LDHA/MCT expression, BA-metabolism phenotype, lipid-metabolism signature) and combinatorial design (metabolic inhibitor + ICI) to maximise effect. In summary, selective targeting of lactate export/production, bile-acid signalling and lipid metabolic machinery offers a refined therapeutic paradigm in CCA—one that exploits tumour metabolic vulnerabilities, engages immune dormancy reversal, and positions CCA for precision immunometabolic therapy.

### 12.6 Multicenter prospective cohorts for standardization and threshold determination of metabolic-immune biomarkers

Establishing multicenter prospective cohorts is essential to validate and standardize metabolic and immune biomarkers in CCA. Such cohorts enable systematic collection of clinical, imaging, omics and outcome data across diverse geographic and ethnic populations, which is critical for determining robust biomarker thresholds for patient stratification, therapeutic monitoring and decision-making. For example, recent metabolomics work in CCA identified serum metabolite signatures associated with recurrence, yet emphasizes the need for larger multicenter validation to define cut-off values that generalize across cohorts (205). Likewise, immunological biomarkers (e.g., CD8^+^ T-cell densities, Treg proportions) have shown prognostic value in single-center studies of intrahepatic CCA, but multicenter data remain scarce (246). Prospective multicenter studies can also address intra- and inter-laboratory variability in sample collection, processing and assay performance—factors that currently limit biomarker reproducibility and clinical implementation. Furthermore, these cohorts can support longitudinal sampling (baseline, on-treatment, post-treatment) to capture dynamic biomarker trajectories, enabling the identification of early response indicators or resistance signatures across metabolic-immune axes. Integration of these data with imaging/radiomics, tissue spatial omics and liquid biopsy enhances stratification and therapeutic adaptation. Importantly, standardizing pre-analytic variables (e.g., timing, biospecimen type, storage), harmonizing multi-omics platforms and agreeing on threshold derivation will allow definition of clinically actionable biomarker cut-offs. In summary, multicenter prospective cohorts provide the infrastructure required to transform promising metabolic-immune biomarker candidates into validated tools for precision medicine in CCA.

### 12.7 Real-time *In vivo* metabolic imaging and tracer strategies in CCA

Real-time metabolic imaging, particularly using PET/MRI and hyperpolarized ^13C-MRI techniques, offers a promising strategy for monitoring metabolic processes in CCA (247). These imaging methods can track key metabolic pathways such as glycolysis (lactate production), glucose metabolism, and fatty acid oxidation (FAO), providing insights into tumor metabolism and immune microenvironment dynamics. For example, hyperpolarized MRI has demonstrated the ability to visualize pyruvate-to-lactate conversion in real time, while PET tracers like ^18F-FDG and ^11C-acetate are used to measure glucose and fatty acid metabolism in tumors (248). In CCA, these technologies could enable early detection of metabolic changes and treatment response, helping stratify patients based on their metabolic-immune phenotypes. Moreover, the combination of metabolic reprogramming and immune modulation is crucial, as metabolites like lactate influence immune cell activity and tumor progression. Future research should focus on clinical validation of these imaging techniques to predict treatment responses and improve patient outcomes in combination metabolic-immunotherapy strategies (39).

## 13 Conclusions

CCA presents a formidable challenge in oncology due to its aggressive nature and limited therapeutic options. Recent advancements have underscored the pivotal role of metabolic reprogramming in tumor progression and immune evasion. The interplay between tumor metabolism and the immune microenvironment offers novel avenues for therapeutic intervention.

Targeting metabolic pathways, such as fatty acid oxidation and glycolysis, has shown promise in preclinical models. For instance, inhibiting fatty acid metabolism has been associated with reduced tumor growth and enhanced immune response. Similarly, modulating glycolytic pathways can influence tumor cell proliferation and immune cell function (91).

The integration of metabolic modulation with ICIs represents a strategic approach to overcome resistance mechanisms inherent in CCA. Combining therapies targeting both metabolic pathways and immune checkpoints has demonstrated synergistic effects, leading to improved therapeutic outcomes (249).

Future research should focus on elucidating the complex interactions between tumor metabolism and the immune system. Developing biomarkers to predict therapeutic responses and identifying optimal combination strategies will be crucial. Additionally, clinical trials evaluating the efficacy and safety of these combined approaches are essential to translate these findings into clinical practice (250).

With the integration of cutting-edge technologies such as single-cell RNA sequencing (scRNA-seq) and ST, a comprehensive understanding of the metabolic-immune axis in CCA is becoming increasingly feasible (125). Recent studies using scRNA-seq and ST have revealed the spatial distribution of immune cells within the TME and their metabolic states, such as glycolysis and fatty acid oxidation, as well as their associations with immune phenotypes, providing mechanistic insights into immune therapy resistance (128). Additionally, emerging research on tumor-associated microbiota has shown that microbial dysbiosis and microbial-derived metabolites play a crucial role in regulating both host metabolism and immunity. Alterations in the gut-liver axis and bile acid metabolism have been closely linked to CCA progression, highlighting the significance of the metabolic-immune-microbiome triad (154).

In conclusion, a comprehensive understanding of the metabolic-immune axis in CCA can inform the development of targeted therapies, offering hope for improved patient outcomes.
